# Treatment of Rheumatoid Arthritis Based on the Inherent Bioactivity of Black Phosphorus Nanosheets

**DOI:** 10.14336/AD.2024.0319

**Published:** 2025-06-03

**Authors:** Cheng Zhuang, Ruiqi Sun, Yuchen Zhang, Qing Zou, Jianxin Zhou, Naijun Dong, Xuyu Zhao, Wenjun Fu, Xiaoke Geng, Jiao Wang, Qian Li, Robert Chunhua Zhao

**Affiliations:** ^1^School of Life Sciences, Shanghai University, Shanghai, China.; ^2^School of Medicine, Shanghai University, Shanghai, China.; ^3^Institute of Basic Medical Sciences Chinese Academy of Medical Sciences, School of Basic Medicine Peking Union Medical College, Beijing, China.; ^4^Center for Excellence in Tissue Engineering, Chinese Academy of Medical Sciences, Beijing, China.; ^5^Beijing Key Laboratory of New Drug Development and Clinical Trial of Stem Cell Therapy (BZ0381), Beijing, China.; ^6^Cell Energy Life Sciences Group Co. LTD, Qingdao, China, 266200.

**Keywords:** Black phosphorus nanosheets, rheumatoid arthritis, autophagy, inflammation, energy metabolism

## Abstract

Rheumatoid arthritis (RA) is an autoimmune disease that affects the living quality of patients, especially the elderly population. RA-related morbidity and mortality increase significantly with age, while current clinical drugs for RA are far from satisfactory and may have serious side effects. Therefore, the development of new drugs with higher biosafety and efficacy is demanding. Black phosphorus nanosheets (BPNSs) have been widely studied because of their excellent biocompatibility. Here, we focus on the inherent bioactivity of BPNSs, report the potential of BPNSs as a therapeutic drug for RA and elucidate the underlying therapeutic mechanism. We find that BPNSs inhibit autophagy at an early stage via the AMPK-mTOR pathway, switch the energy metabolic pathway to oxidative phosphorylation, increase intracellular ATP levels, suppress apoptosis, reduce inflammation and oxidative stress, and down-regulate senescence-associated secretory phenotype (SASP)-related genes in rheumatoid arthritis synovial fibroblasts (RA-SFs). Further, BPNSs induce the apoptosis of macrophages and promote their transition from the M1 to the M2 phenotype by regulating related cytokines. Significantly, the administration of BPNSs can alleviate key pathological features of RA in mice, revealing great therapeutic potential. This study provides a novel option for treating RA, with BPNSs emerging as a promising therapeutic candidate.

## INTRODUCTION

Rheumatoid arthritis (RA) is an autoimmune disease that severely compromises the quality of life of patients. Aging is a significant risk factor for developing RA, with increased morbidity and mortality observed in the elderly population [1]. Therefore, advancing effective RA treatments is crucial for promoting a healthy aging society. The hallmarks of RA are represented by chronic synovitis [2], impaired glucose metabolism [3], abnormally elevated levels of autophagy [4], excessive immune activation [5] and accelerated aging [6]. These features are often interrelated and contribute to the progression of RA [7]. Nonetheless, current clinical drugs for RA are far from satisfactory.

RA medications are commonly classified into disease-modifying antirheumatic drugs (DMARDs) and nonsteroidal anti-inflammatory drugs (NSAIDs). DMARDs can be further categorized into conventional synthetic DMARDs, biologic DMARDs (biologics) and targeted synthetic DMARDs [8]. Among the conventional synthetic DMARDs, methotrexate (MTX), chloroquine (CQ) and hydroxychloroquine (HCQ), represent the primary pharmaceuticals in the treatment of RA [9]. These drugs have been demonstrated to improve joint mobility and inhibit the progression of RA. MTX suppresses immune responses and inflammation mainly by influencing the proliferation, apoptotic events and phenotype of immune cells (reviewed in [10]). The main mechanisms can be summarized as:

1. inhibiting purine and pyrimidine biosynthesis which are required for the proliferation of immune cells,
2. promoting adenosine release and thus stimulating the inhibitory effects of adenosine receptors on inflammatory cells,
3. inducing nitric oxide synthase uncoupling and thus leading to the accumulation of apoptosis-triggering reactive oxygen species (ROS),
4. regulating long intergenic non-coding RNA p21, which is required for p53-medicated apoptosis,
5. suppressing classical pro-inflammatory JAK-STAT and NF-κB signaling pathways,
6. reducing the adherence of neutrophils to fibroblasts [11, 12],
7. inducing the phenotype transition of macrophages from M1 to M2 and thus resulting in the attenuation of pro-inflammatory cytokine expression, osteoclast formation, and bone destruction [13].

CQ and HCQ, both weak bases, tend to accumulate in acidic organelles including lysosomes, endosomes, and the trans-Golgi network, consequently increasing their pH levels. This causes damage to autolysosomes and impedes autophagy, blocks pro-inflammatory cytokine signaling by dampening the activation of endosomal Toll-like receptors and disrupting GMP-AMP synthase signaling [14], and suppresses Ca^2+^-dependent signaling pathways by impairing the Ca^2+^ release process from the endoplasmic reticulum [15]. Additionally, HCQ may inhibit NADPH oxidase [14], a catalyst for ROS production. While ROS can trigger apoptosis, they also function as key upstream signals, triggering the expression and secretion of pro-inflammatory cytokines.

Despite their beneficial effects, the half-life of MTX ranges from hours to days [10, 16, 17], whereas CQ and HCQ persist much longer with half-lives of 40 to 60 days and full elimination times of up to 6 months [18, 19]. This prolonged accumulation greatly increases the risk of side effects, especially retinal toxicity [16, 20]. The common adverse effects of low-dose MTX are gastrointestinal reactions including vomiting and mucosal ulcers, while high-dose MTX may induce life-threatening conditions such as bone marrow suppression and renal failure [21-23]. Regarding CQ and HCQ, the most commonly observed adverse effects include gastrointestinal disturbances, skin discoloration and myotoxicity, as well as rarely-observed but serious cases of retinopathy, neuromuscular toxicity and cardiac myopathy [13]. Biologics and targeted synthetic DMARDs are mainly inhibitors acting on the key players in the inflammatory pathways, such as TNF-receptor antagonists, anti-TNF monoclonal antibodies, IL-6 inhibitors, Janus kinase inhibitors, T-cell co-stimulators, and B-cell depletors [8]. Even though biological DMARDs have increased oral bioavailability and fewer side effects compared with synthetic DMARDs, their high molecular weights and complicated structures greatly limit their delivery efficiency [8].

Common NSAIDs can be classified into carboxylic acids (such as aspirin and ibuprofen), enolic acids (such as meclofenamate) and nonacidic cyclooxygenase-2 selective inhibitors (such as celecoxib) [24]. These drugs are used primarily to alleviate the symptoms of RA, such as inflammation and pain, while not altering the disease process. Therefore, the development of safer and fast-acting anti-RA drugs remains a significant challenge.

**Figure 1.**
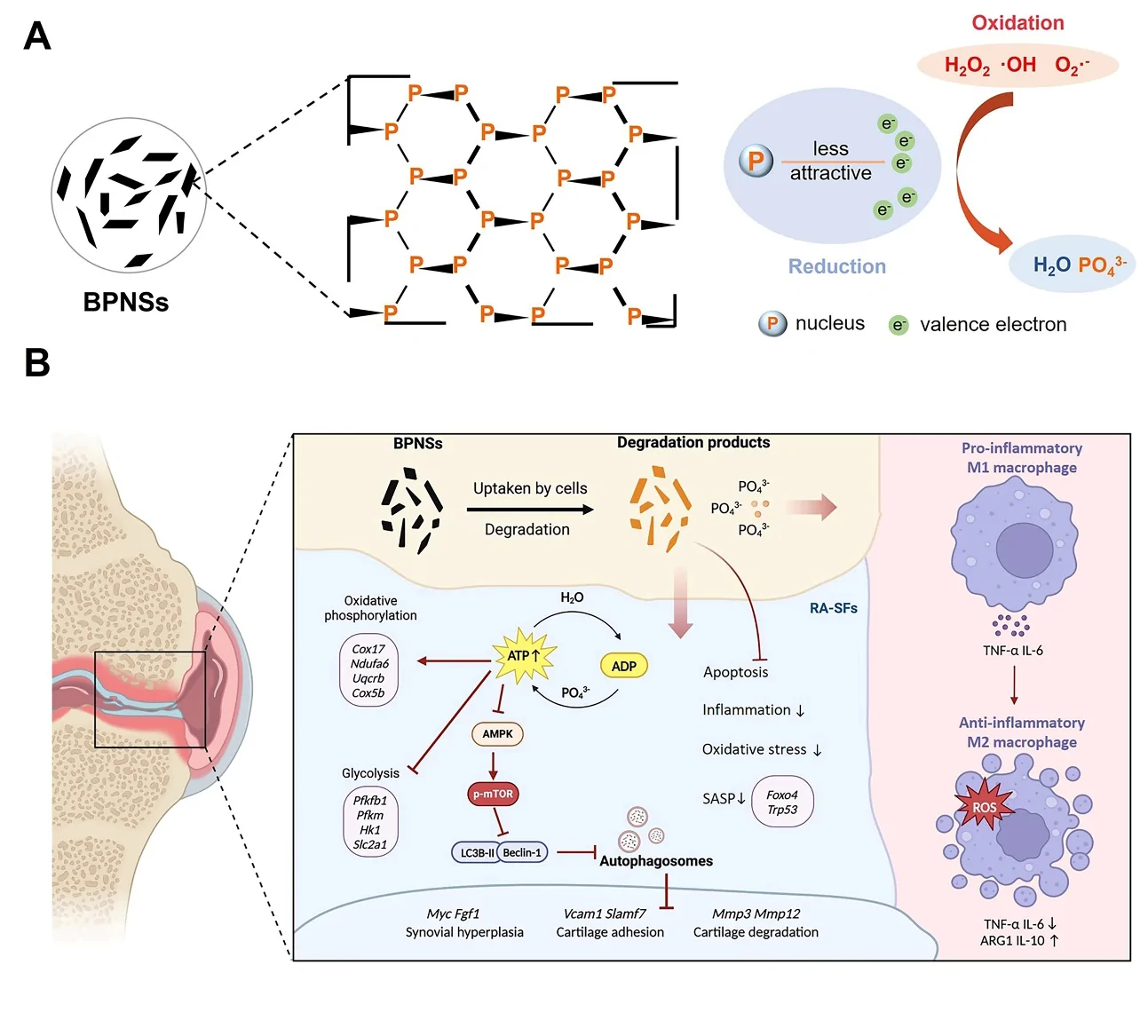
**Schematic illustration demonstrating the chemical structure of BPNSs and their potential mechanism for treating RA**. (**A**) The chemical structure and antioxidant mechanism of BPNSs. BPNSs are pure phosphorus nanomaterials in which the phosphorus atoms are connected by covalent bonds. Phosphorus atoms have five valence electrons. They can either gain three electrons or lose five electrons to form a stable structure of eight electrons. Because of the large radius of phosphorus atoms, the nuclei are less attractive to valence electrons and are more likely to lose electrons. Therefore, when reductive BPNSs encounter oxidative ROS, they neutralize ROS and undergo degradation, producing H_2_O and PO_4_^3-^. (**B**) The treating mechanism of BPNSs for RA as reflected in RA-SFs and macrophages. BPNSs are taken up by RA-SFs and degrade rapidly. The degradation product, PO_4_^3-^, is used for ATP synthesis. Meanwhile, BPNSs restore the metabolic pathway of RA-SFs from aerobic glycolysis to oxidative phosphorylation, resulting in enhanced ATP production. The elevated ATP level signals 'nutrient adequacy' to the cells, which decreases AMPK activity, activating the autophagy inhibitor mTOR and thereby suppressing autophagosome biogenesis. The inhibition of autophagy at an early-stage renders RA-SFs resistant to apoptosis and senescence and hinders the release of pro-inflammatory cytokines and ROS. BPNSs are also taken up by pro-inflammatory M1 macrophages, either inducing their apoptosis or promoting their transition to an anti-inflammatory M2 phenotype. This transition is mediated by suppressing the secretion of TNF-α and IL-6 and promoting the expression of IL-10 and ARG1. The cytoprotective effects of BPNSs on RA-SFs, coupled with their selective inhibition of M1 macrophages contribute to improvements in synovial hyperplasia and cartilage injury.

Black phosphorus nanosheets (BPNSs) have been widely studied because of their excellent biosafety and were initially investigated in the field of anti-tumor research. Yu XF et al. found that BPNSs undergo degradation in lysosomes, resulting in the inhibition of late-stage autophagy, induction of G2/M phase arrest and cell death mediated by autophagy and apoptosis [25]. Based on this study, Shi JL et al. [26] introduced a glycolysis inhibitor 2-deoxy-D-glucose (2DG) on the surface of BPNSs. This induced energy deprivation, further blocking downstream autophagy and compensatory energy supplies, and thereby greatly augmented the therapeutic efficacy of BPNSs against cancer. Moreover, BPNSs exhibit significant potential in photothermal therapy (PTT) and photodynamic therapy (PDT) due to their exceptional photothermal conversion efficiency and controllable band gap [27-29]. According to the Brønsted-Lowry theory [30], the degradation products of BPNSs, phosphate radicals (PO_4_^3-^), display alkalinity and tend to combine with H^+^ in lysosomes to form hydrogen phosphate (HPO_4_^2-^). This reaction results in a reduction in lysosomal acidity and can potentially cause lysosomal dysfunction [26], similar to the mechanism of CQ and HCQ in the treatment of RA [31]. Particularly, BPNSs have a shorter half-life and can be cleared quickly from the body [32]. The electronic structure of phosphorus atoms in BPNSs enables them to effectively donate electrons, neutralizing ROS and thereby acting as antioxidants. These properties render BPNSs a highly biocompatible candidate for RA treatment (Fig. 1A).

Herein, we explore the therapeutic potential of BPNSs for RA. BPNSs demonstrate higher biocompatibility than traditional RA drugs. For rheumatoid arthritis synovial fibroblasts (RA-SFs), BPNSs exert cytoprotective effects by inhibiting early-stage autophagy through the regulation of AMP-activated protein kinase-mammalian target of rapamycin (AMPK-mTOR) pathway, increasing intracellular ATP levels via the restoration of oxidative phosphorylation, resisting apoptosis, suppressing inflammation and oxidative stress, as well as down-regulating senescence-associated secretory phenotype (SASP)-related genes (Fig. 1B). For macrophages, BPNSs show selective toxicity towards the pro-inflammatory M1 phenotype and promote the anti-inflammatory M2 phenotype by regulating related cytokines (Fig. 1B). Significantly, the therapeutic effects of BPNSs against synovial hyperplasia and cartilage damage were confirmed in an RA mouse model, highlighting their potential as a promising clinical drug for RA.

## MATERIALS AND METHODS

### Material

The black phosphorus (BP) crystal powder was purchased from XFNANO (China). N-methyl-2-pyrrolidone (NMP) and 30% H_2_O_2_ were purchased from Sinopharm Chemical Reagent Co., Ltd. Hydroxychloroquine sulfate (HCQ) and methotrexate disodium salt (MTX) were obtained from Bide Pharmatech Co., Ltd. and Sigma-Aldrich respectively. Chloroquine phosphate (CQ) was purchased from Shanghai Aladdin Biochemical Technology Co., Ltd. Annexin V-FITC/PI Apoptosis Detection Kit and Cell Counting Kit-8 (CCK-8) were bought from Yeasen Biotechnology (Shanghai) Co., Ltd. Enhanced ATP Assay Kit, Malachite Green Phosphate Detection Kit, adenovirus Ad-mCherry-GFP-LC3B, Hydrogen Peroxide Assay Kit and Total Superoxide Dismutase Assay Kit with WST-8 were purchased from Beyotime Biotechnology. Phosphate buffered saline (PBS, pH 7.4), fetal bovine serum (FBS), tris buffered saline (TBS), DMEM (high glucose), trypsin, penicillin-streptomycin and TRIzol were supplied by Thermo Fisher Scientific. Granulocyte-macrophage colony stimulating factor (GM-CSF) and all antibodies were purchased from ABclonal Technology Co., Ltd. Lipolyaccharide (LPS), collagenase IV, RIPA lysis buffer, rapid blocking buffer and ROS assay kit were purchased from Wuhan Servicebio Technology Co., Ltd. ChamQ SYBR qPCR master mix (without ROX), HiScript III RT supermix for qPCR (+ gDNA wiper) and BCA protein quantification kit were purchased from Vazyme Biotech Co., Ltd. The CheKine™ Micro Lactate Assay Kit was purchased from Abbkine Scientific Co., Ltd. All other analytical reagents were purchased from Sinopharm Chemical Reagent Co., Ltd. and used without further purification.

### Synthesis of BPNSs

BPNSs were prepared through the liquid exfoliation method [33]. Briefly, 20 mg of BP crystal powder was dispersed in 20 mL of NMP. The mixture was incubated in an ice bath with an ultrasonic crusher (JY92-IIN, SCIENTZ) for 12 h (working power: 60%, On/Off cycle: 4 s/4 s). Afterwards, the BP dispersion liquid was centrifuged at 5000 rpm at 4°C for 10 min to remove oversized BP particles. BPNSs were obtained by centrifugation at 12000 rpm and 4°C. The nanoparticles were stored in newly prepared NMP and rinsed with PBS before subsequent experiments.

### Synthesis of HCeO_2_ NPs

SiO_2_ nanoparticles (SiO_2_ NPs) were first prepared as a template. 50 mL of ethanol and 1 mL of deionized water were mixed in a flask and heated to 40°C. Next, 1.5 mL of tetraethyl orthosilicate (TEOS) and 2.4 mL of ammonia solution (25%-28%) were added to the mixture followed by stirring at 200 rpm for 12 h. After stirring, SiO_2_ NP_S_ were collected by centrifugation at 7000 rpm for 5 min, washed twice with deionized water and anhydrous ethanol each and then dried in a vacuum oven at 60°C. 0.1 g of SiO_2_ NPs were dispersed in 45 mL of deionized water and heated to 90°C. 0.2 g of Ce(NO_3_)_3_·6H_2_O and 0.09 g of hexamethylenetetramine (HMTA) were dissolved in 2.5 mL of deionized water respectively. The Ce(NO_3_)_3_·6H_2_O solution and the HMTA solution were added to the SiO_2_ NPs dispersion liquid, followed by stirring at 1500 rpm for 3 h. After stirring, the reaction products were collected following the same steps as for collecting SiO_2_ NPs described above. Then, the products were heated from room temperature with a heating rate of 10°C/min and maintained at 600 °C for 2 h. The calcined products were dispersed in a NaOH solution (0.5 M) for 6 h at room temperature while stirring at 800 rpm to remove SiO_2_. Afterwards, HCeO_2_ NPs were collected by centrifugation at 5000 rpm, washed with deionized water 3 times and dried in a vacuum oven at 60°C.

### Characterization of nanomaterials

Transmission electron microscopy (TEM) and atomic force microscopy (AFM) were employed to determine the size and thickness of nanoparticles, using a transmission electron microscope (JEM-2100F, JEOL Ltd.) and an atomic force microscope (AP-0190, Park Systems), respectively. The Raman spectrum was acquired with an inVia Raman microscope (Renishaw). Dynamic Light Scattering (DLS) was applied to characterize the average size of BPNSs. Zeta potentials were measured in water (pH 7.0) using Zetasizer Nano ZSE (Malvern). The X-ray diffraction spectrum of HCeO_2_ NPs was obtained with an X-ray diffractometer (18KW, D-MAX 2500, Rigaku) to determine their composition and crystalline structure.

### Animals

Male C57BL/6 mice (6-8 weeks) were purchased from Shanghai JieSiJie Laboratory Animal Co., Ltd. and handled in accordance with the international animal care guidelines. All animals were kept on a 12/12 h light/dark cycle, at a constant temperature (22±1°C) and humidity (60-80%), with food and water *ad libitum*. The animal protocols were approved by the Animal Ethics Committee of Shanghai University (ECSHU 2023-004).

### Determination of ROS scavenging capacity

A Hydrogen Peroxide Assay Kit was used to determine the H_2_O_2_ scavenging capacity of BPNSs and HCeO_2_ NPs. Briefly, H_2_O_2_ oxidizes Fe^2+^ to Fe^3+^, which reacts with xylenol orange (XO) to generate a purple complex with a maximum absorption at 560 nm. The initial concentration of H_2_O_2_ was 30 mM. The concentrations of BPNSs tested were 1, 5, 25, 120 μg/mL and those of HCeO_2_ NPs were 5, 10, 25, 125 μg/mL. The absorbance values at 560 nm were measured with an ultraviolet spectrophotometer (NanoDrop 8000, Thermo Fisher Scientific) to indicate the concentration of H_2_O_2_.

A Total Superoxide Dismutase Assay Kit with WST-8 was used to measure the O_2_·^-^ scavenging capacity of BPNSs and HCeO_2_ NPs. O_2_·^-^, derived from xanthine under the catalysis of xanthine oxidase, can react with WST-8 to generate the formazan dye with an absorption at 450 nm. Therefore, the absorbance values at 450 nm were negatively correlated with the O_2_·^-^ scavenging capacities of the nanoparticles.

·OH was generated via the Fenton reaction of Fe^2+^ (10 mM) and H_2_O_2_ (5 mM), with methylene blue (MB, 50 μg/mL) employed as the indicator of ·OH. The absorbance values at 660 nm were recorded to calculate the clearing efficiencies of ·OH by BPNSs and HCeO_2_ NPs.

### Cell isolation and culture

Primary mouse synovial fibroblasts (SFs) [34] and mouse bone marrow-derived macrophages (BMDMs) were isolated from C57BL/6 mice. The mice were anesthetized with 1.25% tribromoethanol (250 mg/kg) and sacrificed by cervical dislocation. The hind limb skin of mice was cut with surgical scissors and tweezers, and the synovium (white or yellowish and spongy) around the hip joint was isolated and transferred to a petri dish containing DMEM. The synovium was sheared into pieces (about 1 mm^3^ in size) and digested with 1 mL of collagenase IV solution (5 mg/mL) at 37° with shaking (200 times/min) for 1 h. Then the digestion mixture vibrated at high speed for 90 s and filtered with a 70 μm cell strainer. The cells were collected by centrifugation at 300 g for 5 min and cultured in DMEM containing 15% FBS and 1% penicillin-streptomycin.

To obtain primary BMDMs, the femur and tibia were isolated from mice, and sheared on both sides to expose the marrow cavity. The marrow cavity was rinsed with DMEM repeatedly until the femur and tibia turned pale. The bone marrow mass was collected and dispersed with a pipette, and centrifuged at 1000 rpm for 5 min. The cell pellets were resuspended and lysed in red blood cell lysis buffer for 5 min. Then the cell suspension was centrifuged and washed with culture medium (DMEM supplemented with 20% FBS, 1% penicillin-streptomycin and 40 ng/mL GM-CSF) twice. Finally, the cells were equally seeded in T25 flasks (6 × 10^6^ cells per flask) or 6-well plates (2 × 10^6^ cells per well) and cultured for 1 week to obtain mature BMDMs.

### Measurement of cell viability

The cytotoxicity of BPNSs on SFs and BMDMs was evaluated by measuring the cell viability with a CCK-8 assay kit. In brief, SFs and BMDMs were seeded in 96-well plates at a density of 5000 cells per well and cultured for 24 h. BPNSs were prepared in culture media with concentrations ranging from 0 to 200 μg/mL (0, 5, 10, 50, 100, 200 μg/mL) and from 0 to 100 μg/mL (0, 5, 10, 25, 50, 100 μg/mL), respectively. The concentrations of CQ, HCQ and MTX ranged from 0 to 200 μg/mL. The absorbance values were measured at 450 nm using a multifunctional microplate reader (VICTOR X3, PerkinElmer).

### Establishment of inflammatory cell models and BPNSs treatment

LPS was used to construct inflammatory cell models. SFs were treated with LPS (2 μg/mL) for 24 h to generate RA-SFs. BMDMs were treated with LPS (200 ng/mL) for 4 h to induce the M1 phenotype. The treatment concentration of BPNSs was 5 μg/mL. Untreated cells cultured under the same conditions were used as negative controls (NC).

### Determination of oxidative stress levels in vitro

SFs and BMDMs were seeded in 12-well plates and treated with LPS as described above. The culture media were removed, and the cells were rinsed three times with PBS. Then 500 μL of 2',7'-dichlorodihydrofluorescein diacetate (DCFH-DA) working solution was added to each well and incubated with the cells in dark at 37°C for 30 min. After the incubation, the cells were washed with PBS and imaged with a fluorescence microscope (MF53-N, MShot). The average fluorescence intensity of DCF (2',7'-dichlorofluorescein) labeling was quantified with Image J to calculate the ROS level, indicating the oxidative stress level in each cell group.

### Measurement of intracellular ATP levels

The ATP levels were measured with an ATP assay kit based on the conversion of luciferin into oxyluciferin by firefly luciferase. SFs were seeded in a 12-well plate (1 × 10^5^ cells per well) and induced by LPS, followed by BPNSs treatment. Cells were rinsed with PBS and lysed in lysis buffer on ice. The lysate was centrifuged at 12000 rpm at 4°. The supernatant was collected and mixed with ATP test working solution according to the manufacturer’s instructions. The chemiluminescence was measured at 560 nm with a multifunctional microplate reader (VICTOR X3, PerkinElmer).

### Determination of lactate levels

SFs were seeded in a 6-well plate (3 × 10^5^ cells per well) and induced by LPS, followed by BPNSs treatment. The culture media were collected and a CheKine™ Micro Lactate Assay Kit was employed to measure the extracellular lactate levels according to the manufacturer’s instructions.

### Determination of the degradation rate of BPNSs

The phosphate radical concentration was measured to indicate the degradation rate of BPNSs (20 μg/mL) in water and H_2_O_2_ (5 mM) with a Malachite Green Phosphate Detection Kit. SFs (1.2 × 10^5^ cells per well) were seeded in 12-well plates. After the treatment of LPS and BPNSs, the culture media were removed, and SFs were rinsed three times with saline to remove BPNSs that adhered to the cell surface. The cells were lysed with the cell lysis buffer (200 μL per well) containing NaOH (0.5 M) and 0.5% TritonX-100 for 8 min. Then the cell lysate was centrifuged at 12000 rpm at 4°C for 5 min. The supernatant was collected to determine the phosphate radical level.

### ELISA quantification of immune regulatory proteins

SFs (1.2 × 10^5^ cells per well) and BMDMs (at the same density per well) were seeded in 12-well plates, respectively. After the induction of LPS and the treatment of BPNSs, the culture media were collected for enzyme linked immunosorbent assay (ELISA) to determine the levels of secreted proteins. For SFs, the levels of TNF-α and IL-1β were measured. IL-6, TNF-α, ARG1 (Arginase 1) and IL-10 were evaluated for BMDMs.

### Quantitative real-time PCR (qRT-PCR)

Total RNA of SFs and BMDMs was extracted with TRIzol. The RNA concentration was measured with NanoPhotometer C40 (IMPLEN). Then cDNA was synthesized with 1 μg of total RNA and diluted 10 times with ddH_2_O. The qPCR reaction mixture was prepared with 10 µL of 2 × ChamQ SYBR qPCR master mix (without ROX), 0.4 µL of each primer (10 μM) and 9.2 µL of diluted cDNA. Real-time PCR was carried out using PrimePCR™ Analysis Software (Bio-Rad) with the following program: 95° for 3 min; 40 cycles of 95° for 5 s, 60° for 30 s; 95° for 5 s; 60 repeats of an increment of 0.5° from 65° to 95°, 5s for each repeat. The primers used were listed in Table 1.

**Table 1:** Genes and primer sequences for qRT-PCR.

Gene name	Primer sequence (5’→3’)
***Arg1-F***	CTCCAAGCCAAAGTCCTTAGAG
***Arg1-R***	AGGAGCTGTCATTAGGGACATC
***Il-1β-F***	GCACTACAGGCTCCGAGATGAAC
***Il-1β-R***	TTGTCGTTGCTTGGTTCTCCTTGT
***Tnf-α-F***	TACTGAACTTCGGGGTGATTGGTCC
***Tnf-α-R***	CAGCCTTGTCCCTTGAAGAGAAC
***Il-6-F***	CCGGAGAGGAGACTTCACAG
***Il-6-R*** ***Il-10-F*** ***Il-10-R*** ***β-actin-F*** ***β-actin-R*** ***caspase-3-F*** ***caspase-3-R***	GGAAATTGGGGTAGGAAGGA GCTCTTACTGACTGGCATGAG CGCAGCTCTAGGAGCATGTG CGTTGACATCCGTAAAGACC AACAGTCCGCCTAGAAGCAC CTGATGAGGAGATGGCTTG ACCCGTCCTTTGAATTTCT

### Western blotting

Total proteins were extracted from SFs with RIPA lysis buffer and the protein concentration was measured with a BCA protein quantification kit. Then proteins were mixed with SDS-PAGE sample buffer and denatured at 95°C for 10 min. After the electrophoresis, the proteins were transferred to nitrocellulose membranes, followed by blocking with rapid blocking buffer for 30 min. Then primary antibodies against LC3B (ABclonal, A7198), P62 (ABclonal, A19700), Beclin-1(ABclonal, A7353), caspase-3 (ABclonal, A2156), BAX (ABclonal, A0207), BCL-2 (ABclonal, A0208), mTOR (ABclonal, A2445), p-mTOR (ABclonal, AP0115), AMPK (ABclonal, A1229), p-AMPK (ABclonal, AP0871), IL-1β (ABclonal, A16288), β-actin (ABclonal, AC004), GAPDH (ABclonal, AC002), β-tubulin (ABclonal, AC008) or vinculin (ABclonal, A2752) were incubated with the membranes at 4°C overnight. Subsequently, membranes were washed with TBST (0.2% Triton X-100 in TBS) 3 times and incubated with fluorescent secondary antibodies at room temperature for 50 min. After another 3 washes with PBS, membranes were visualized using an iBright imaging system (Thermo Fisher Scientific). The intensities of protein bands were quantified with Image J.

### Evaluation of apoptosis

BMDMs were seeded in 6-well plates at the same density per well. After the treatment of LPS and BPNSs, cells were digested with trypsin, collected by centrifugation at 1200 rpm for 4 min, and rinsed twice with cold PBS. The cell pellets were resuspended in 100 μL of binding buffer. Then 5 μL of Annexin V-FITC and 10 μL of PI staining solutions were added into the cell suspension in sequence. The mixture was kept in dark at room temperature for 15 min. After the staining, 400 μL of binding buffer was added and the cells were quantified with a flow cytometer (Cytoflex).

### Tracking of the autophagic process

SFs were seeded in an 8 chambered coverglass at a density of 2 × 10^4^ cells per well and treated as described above. The culture media were replaced with fresh media containing the adenovirus expressing mCherry-GFP-LC3B fusion protein (Ad-mCherry-GFP-LC3B) (with an MOI of 150) and incubated with the cells at 37°C for 24 h. Then the media were replaced with fresh media and the cells were further incubated at 37°C for another 24 h. LC3B puncta were viewed and photographed with a Laser scanning confocal microscope (FV3000, Olympus). The red and green dots were quantified with Image J according to the manufacturer’s instructions.

### The establishment of collagen-induced arthritis (CIA) model

Collagen II (2 mg/mL) was dissolved in 0.1 M acetic acid with continuous stirring at 4°C and the mixture was incubated at 4°C overnight. Meanwhile, inactivated Bacillus Calmette Guerin (BCG) vaccine (2 mg/mL) was mixed with paraffin to prepare complete Freund’s adjuvant (CFA) [35]. Then the collagen II solution and CFA were mixed in equal volumes to prepare a collagen II emulsion. 6-8 weeks old male mice were injected intradermally with 0.1 mL of emulsion at the base of the tail. On day 21, a second dose of 0.1 mL emulsion was injected intraperitoneally. The RA symptoms of CIA mice were evaluated by scoring the degree of paw swelling according to the following criterion: 0, no swelling; 1, mild swelling of individual toes; 2, moderate swelling of individual toes; 3, severe swelling of whole paws; 4, extreme swelling of limbs. The mice with a score of 3 were selected for the following experiments.

### Biosecurity assessment of BPNSs in vivo

Toxicological analysis was performed in C57BL/6 mice (6-8 weeks, about 25 g). The mice were divided into three groups randomly (n = 5): untreated, treated with PBS or BPNSs. BPNSs (5 mg/kg) were administrated by tail vein injection. After 24 h post injection, the mice were sacrificed. Major organs, including the heart, liver, spleen, lungs, and kidneys, were collected and fixed in 4% paraformaldehyde (PFA) for hematoxylin and eosin (H&E) staining to evaluate histological changes. Meanwhile, the whole blood was collected to obtain serum samples which were used for determining routine blood parameters (n = 3).

### Treatment of CIA mice with BPNSs

The CIA mice with a paw swelling score of 3 were randomly divided into 4 groups depending on the dosage of BPNSs (n = 6): PBS, BPNSs + (0.2 mg/kg), BPNSs ++ (0.5 mg/kg) and BPNSs +++ (1.2 mg/kg). Each group received intra-articular injections every three days for a total of 4 times. The changes in arthritis index were observed and recorded for each group. The mice were sacrificed on day 51 and their ankle joints were collected for histological analysis.

### Histological staining

After different treatments, mouse ankle joints were collected and fixed in 4% PFA for 3 days. Then the ankle joints were decalcified in decalcifying solution (Serivcebio) at room temperature for 14 days, embedded in paraffin and cut to obtain 3 μm sections. The tissue sections were incubated with antibodies against TNF-α (ABclonal, A11534) or IL-1β (ABclonal, A16288) followed by secondary antibody staining with goat anti-rabbit Alexa Fluor 594 (Abcam, Ab150080). Subsequently, the sections were mounted with antifade mounting medium containing DAPI. For 3,3′-Diaminobenzidine (DAB) staining, the tissue sections were incubated with antibodies against caspase-3 (ABclonal, A2156), Beclin-1 (ABclonal, A21695), GLUT1 (glucose transporter 1, ABclonal, A6982) or P21 (ABclonal, A19094) followed by secondary antibody staining with S-vision poly-HRP conjugated Goat Anti-Rabbit IgG (H + L) (Servicebio). The tissue sections were sealed after hematoxylin staining. Meanwhile, H&E staining and Safranin-O (Saf-O) staining were conducted to evaluate synovial hyperplasia and cartilage injury, respectively. All images were acquired with a fluorescence microscope (MF53-N, MShot). The fluorescence intensity, target protein-positive area, and cartilage content were quantified with Image J.

### Statistical analysis

All quantitative data are shown as mean ± SEM in the figures. The normality of each dataset was assessed with the Shapiro-Wilk test. All datasets used to determine the significance of group differences followed a normal distribution. One-way ANOVA with Tukey's *post hoc* test was performed for three or more groups and Student’s t-test for two groups to determine the significance of differences between groups. Significance levels were set at *p < 0.05, **p < 0.01, ***p < 0.001, and ****p < 0.0001. The data were analyzed, and the statistical graphs were drawn with GraphPad Prism 9.0.

**Figure 2.**
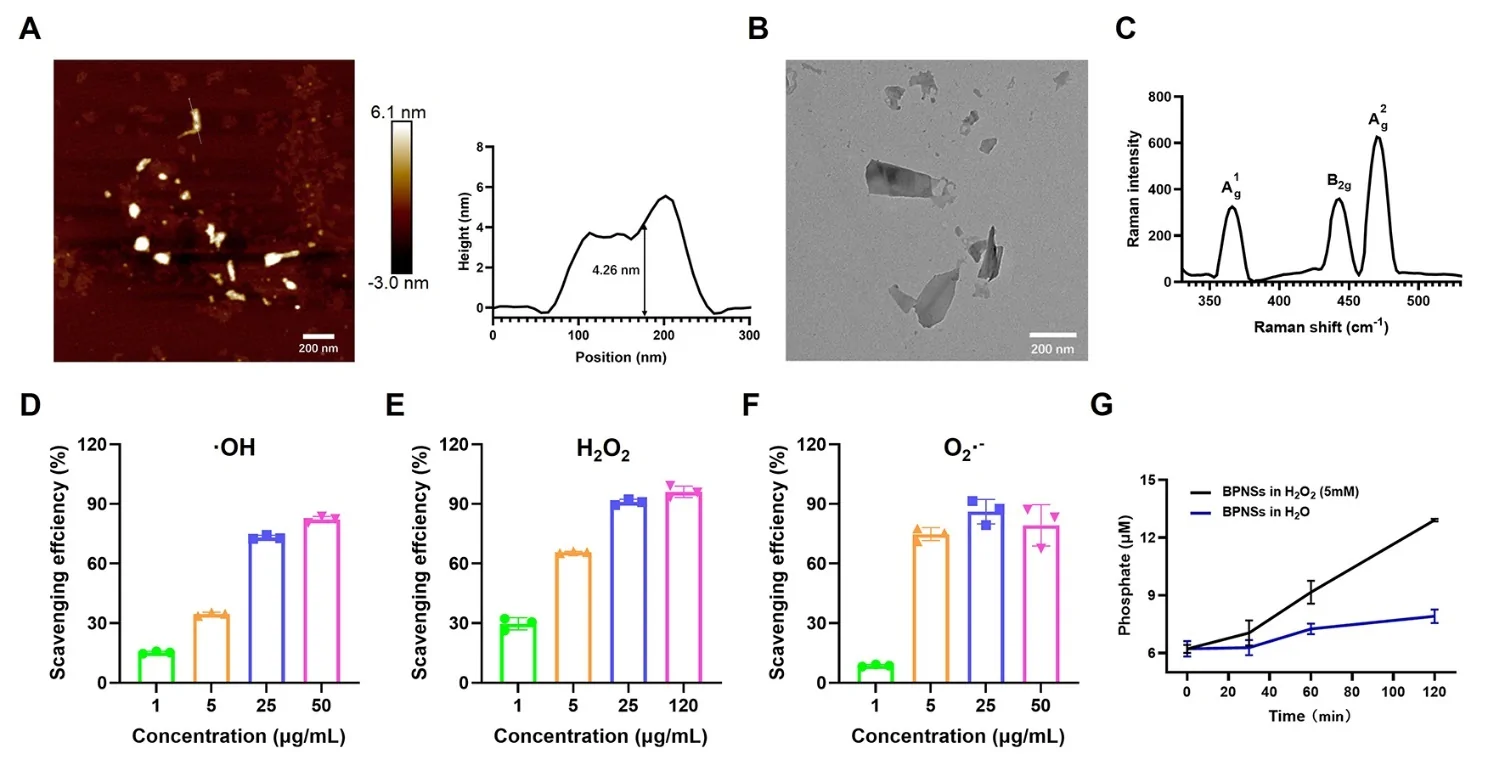
**Characterization of BPNSs**. (**A**) A representative AFM image and corresponding height analysis of BPNSs. Scale bar, 200 nm. (**B**) A representative TEM image of BPNSs. Scale bar, 200 nm. (**C**) The Raman scattering spectrum of BPNSs. (**D-F**) The scavenging capacities of BPNSs for (**D**) ·OH, (**E**) H_2_O_2_ and (**F**) O_2_·^-^. (**G**) Degradation behavior of BPNSs in H_2_O and H_2_O_2_, respectively. For (**D-G**), n=3 independent experiments, indicating 3 biological replicates.

## RESULTS

### Characterization and antioxidant capacity evaluation of BPNSs

The average size and thickness of BPNSs were determined to be 200 nm and 4.5 nm, respectively, by AFM and TEM (Fig. 2A and 2B). In the Raman scattering spectrum, peaks observed at 366.06 cm^-1^, 442.76 cm^-1^ and 470.93 cm^-1^ corresponded to A^1^_g_, B_2g_ and A^2^_g_, representing the characteristic peaks of BPNSs (Fig. 2C). Using DLS, the hydrodynamic diameter and zeta potential of BPNSs were measured to be 335.9 ±11.2 nm and -52.93 ± 1.03 mV respectively (Supplementary Fig. 1).

Since oxidative stress is one of the major factors contributing to the development of RA symptoms, we next evaluated the antioxidant capacity of BPNSs in scavenging ·OH, H_2_O_2_, and O_2_·^-^. As shown in Fig. 2D, the scavenging rate for ·OH by BPNSs increased with their concentration increasing from 1 μg/mL to 50 μg/mL, reaching 73.19% at 25 μg/mL. The scavenging efficiency of 30 mM H_2_O_2_ by 25 μg/mL BPNSs was determined to be 90.92% (Fig. 2E). Similarly, the scavenging rate of 25 μg/mL BPNSs for O_2_·^-^ reached 86.12% (Fig. 2F). These observations indicate that BPNSs have good antioxidant capacity. Meanwhile, we synthesized HCeO_2_ NPs (Supplementary Fig. 2A-C), another emerging nanomaterial with antioxidant properties and potential for RA treatment [36, 37]. Under the same conditions, the scavenging rates of 25 μg/mL HCeO_2_ NPs for ·OH, H_2_O_2_, and O_2_·^-^ were 9.01%, 27.78% and 14.92%, respectively (Supplementary Fig. 2D-F). This demonstrates that the antioxidant capacity of HCeO_2_ NPs is much lower compared to BPNSs.

In addition, we monitored the degradation behaviors of BPNSs in H_2_O and 5 mM H_2_O_2_ separately. It was found that the phosphate concentrations in both solutions increased in a time-dependent manner and were significantly higher in H_2_O_2_ than in H_2_O (Fig. 2G). This makes BPNSs promising for use in the context of inflammatory lesions, as their fast decomposition greatly reduces the risk of prolonged retention within the organism.

### Comparison of the effects of BPNSs and clinical drugs for RA on the cell viability of SFs

**Figure 3.**
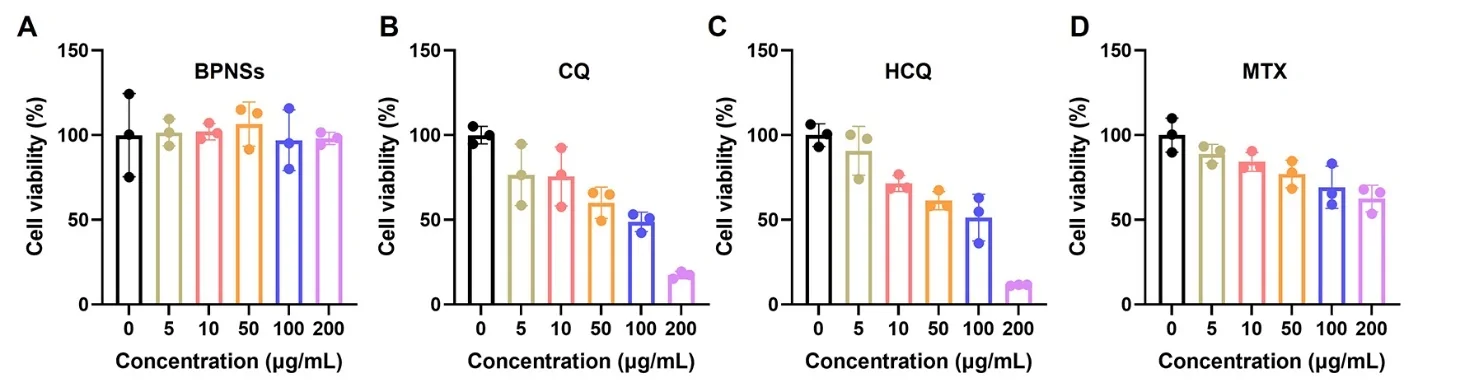
**The viability of SFs following the treatments of (A) BPNSs, (B) CQ, (C) HCQ and (D) MTX**. The values were quantified with a CCK-8 kit from 3 batches of cells as 3 biological replicates (n=3).

To evaluate the biosafety of BPNSs, their toxicity on normal primary SFs isolated from mouse hip joint synovium [34] was assessed. At a concentration of 200 μg/mL, a notably high concentration compared with other nanomedicines, BPNSs generated minimal effects on the cell viability of SFs (Fig. 3A). Whereas, the three clinical drugs for RA (CQ, HCQ, MTX) all decreased the cell viability of SFs in a concentration-dependent manner (Fig. 3B-3D), likely due to their long half-lives and tendency to accumulate [38]. By contrast, BPNSs are known to have a shorter half-life [32], contributing to their favorable biocompatibility.

### BPNSs inhibit autophagy at an early stage and raise the ATP level in RA-SFs

BPNSs may inhibit autophagy at a late stage in cancer cells [26], similar to the mechanism of CQ and HCQ in the treatment of RA [39]. Given that overactivated autophagy is commonly seen in SFs in the context of RA [40], we investigated whether BPNSs could alleviate this pathology. RA-SFs were established by the induction of LPS (2 μg/mL, 24 h). We tracked the autophagic process with an adenovirus (Ad-mCherry-GFP-LC3B) expressing the autophagosome marker protein LC3B [41]. At the late stage of autophagy when autophagosomes fuse with lysosomes, the acidic environment inside lysosomes quenches the green fluorescence (GFP), and consequently, autolysosomes are labeled with only the red fluorescence (mCherry). Compared with normal SFs, we found significant increases in the numbers of both green and red dots in RA-SFs, which were decreased following BPNSs treatment (Supplementary Fig. 3A and 3B). However, there was no obvious difference in the ratio of green to red dots between untreated and BPNSs-treated RA-SFs (Supplementary Fig. 3A and 3C). These observations suggest that BPNSs could inhibit autophagy in RA-SFs, although this effect was not evident during the late stage of autophagy.

**Figure 4.**
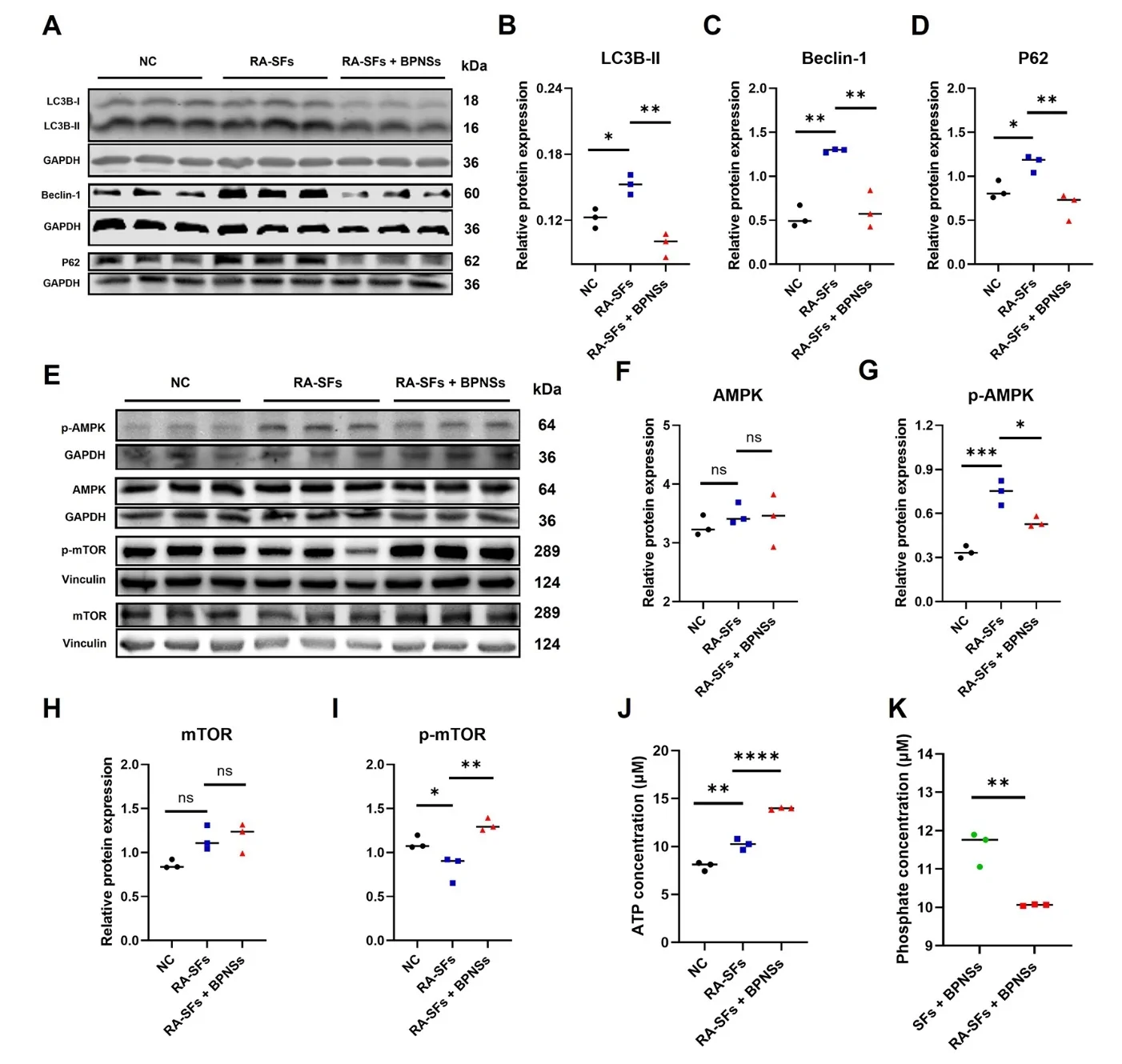
**BPNSs inhibit autophagy at an early stage and raise the ATP level in RA-SFs**. (**A**) Representative western blots and (B-D) quantification of LC3B-II, Beclin-1 and P62 in control cells (NC), RA-SFs and BPNSs-treated RA-SFs. (**E**) Representative western blots and (F-I) quantification of total AMPK, p-AMPK, total mTOR and p-mTOR in control cells (NC), RA-SFs and BPNSs-treated RA-SFs. (**J**) The levels of ATP in control cells (NC), RA-SFs and BPNSs-treated RA-SFs. (**K**) The levels of phosphate in SFs or RA-SFs following BPNSs treatment. GAPDH or vinculin were used as the loading controls for western blotting experiments. N=3 batches of cells per group, indicating 3 biological replicates. The normality of data was evaluated with the Shapiro-Wilk test. Significance of difference was determined through one-way ANOVA with Tukey's *post hoc* test for (B-D, F-J) or Student’s t-test for (K). Significance levels are denoted as *p < 0.05, **p < 0.01, ***p < 0.001, ****p < 0.0001, and ^ns^p: not significant. NC, negative control, normal SFs without LPS induction; p-AMPK, phosphorylated AMPK; p-mTOR, phosphorylated mTOR. The phosphorylation of AMPK and mTOR indicates their activation.

Significantly, the levels of LC3B-II, the lipidated isoform of LC3B and a crucial marker of autophagosomes [42], as well as Beclin-1, an important regulator of autophagy initiation [43], were both decreased in RA-SFs treated with BPNSs (5 μg/mL, 24 h) (Fig. 4A-C). Accordingly, the activation of AMPK, a key initiator of autophagy [44] was significantly diminished in RA-SFs upon BPNSs treatment (Fig. 4E-G). Conversely, mTOR, an autophagy inhibitor typically suppressed by AMPK [45-47], was activated by BPNSs (Fig. 4E, 4H and 4I). These results demonstrate that BPNSs inhibit autophagy at an early stage. Furthermore, AMPK serves as a crucial sensor of cellular energy levels and can be inhibited by high ATP levels [45, 47, 48]. Consistently, BPNSs treatment significantly elevated the ATP level in RA-SFs (Fig. 4J). This observed ATP increase may be attributed to the degradation product of BPNSs, PO_4_^3-^, which actively participates in the synthesis of ATP [49]. Supporting this, the phosphate concentration in BPNSs-treated RA-SFs was lower than in normal SFs treated with BPNSs (Fig. 4K), suggesting that under inflammatory conditions, BNPSs contribute to the conversion of inorganic phosphorus to organophosphorus.

### BPNSs suppress apoptosis, inflammation, and oxidative stress in RA-SFs

**Figure 5.**
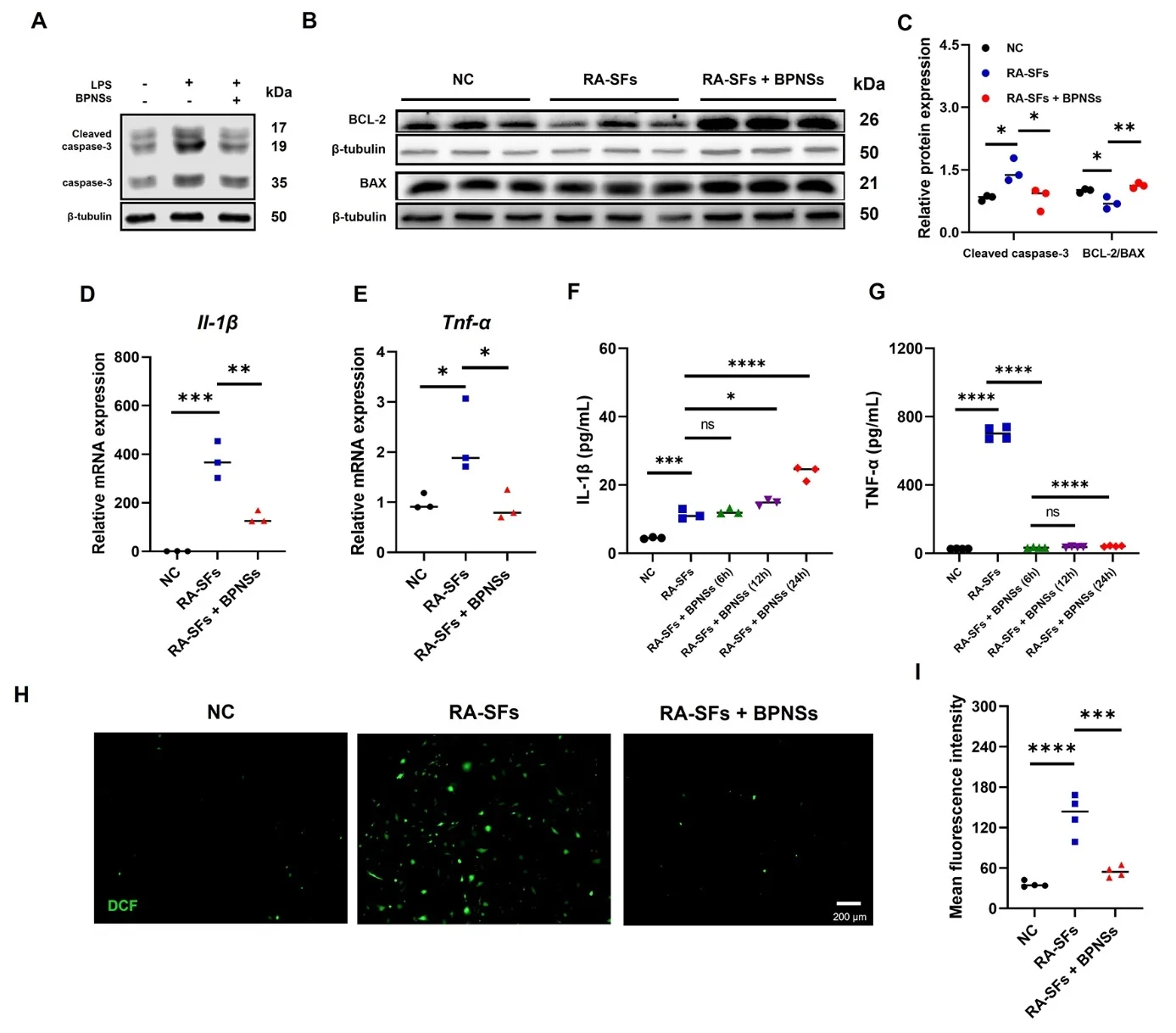
**BPNSs protect RA-SFs against apoptosis, inflammation, and oxidative stress**. (A and B) Representative western blots of total caspase-3, cleaved caspase-3, BCL-2 and BAX in control cells (NC), RA-SFs and BPNSs-treated RA-SFs. The levels of cleaved caspase-3 indicate the extent of caspase-3 activation. β-tubulin was used as the loading control. (**C**) Quantification of cleaved caspase-3 and the ratio of BCL-2/BAX represented by (A) and (B). (D and E) The relative mRNA expression levels of *Il-1β* and *Tnf-α* in control cells (NC), RA-SFs and BPNSs-treated RA-SFs. (F and G) Quantification of IL-1β and TNF-α released from control cells (NC), RA-SFs and BPNSs-treated RA-SFs. For the BPNSs-treated group, the culture media were collected for ELISA analysis at 6 h, 12 h and 24 h after initiating BPNSs treatment. (**H**) Representative immunofluorescence images and (I) quantification of intracellular oxidative stress levels in control cells (NC), RA-SFs and BPNSs-treated RA-SFs. The oxidative stress level is represented by the average fluorescence intensity of DCF labeling. Scale bar, 200 μm. For (C-F), n=3 batches of cells per group, indicating 3 biological replicates. For (G and I), n=4 batches of cells per group, indicating 4 biological replicates. The normality of data was evaluated with the Shapiro-Wilk test. Significance of difference was determined through one-way ANOVA with Tukey's *post hoc* test. Significance levels are denoted as *p < 0.05, **p < 0.01, ***p < 0.001, ****p < 0.0001 and ^ns^p: not significant. NC, negative control, normal SFs without LPS induction.

Give the interplay between autophagy and apoptosis in the regulation of cell survival and death, we next evaluated the effects of BPNSs-induced autophagy inhibition on apoptosis. A decrease in the activation of the apoptosis executor caspase-3, indicated by reduced levels of cleaved caspase-3, was observed in RA-SFs after BPNSs treatment (Fig. 5A and 5C). Meanwhile, the ratio of anti-apoptotic BCL-2 to pro-apoptotic BAX was significantly increased in BPNSs-treated RA-SFs (Fig. 5B and 5C). These observations suggest that the suppression of BPNSs on autophagy in RA-SFs may drive the cell fate away from apoptosis, supporting the cytoprotective effects of BPNSs [50].

Interestingly, the expression of P62 (also known as sequestosome-1, SQSTM1) was elevated in RA-SFs compared to normal SFs and decreased after the treatment of BPNSs (Fig. 4A and 4D). This seems to contradict previous research, as P62 is selectively degraded in the process of autophagy [51]. However, P62 has also been reported to activate the NF-κB pathway [52, 53]. Indeed, the expression levels of pro-inflammatory cytokines TNF-α and IL-1β were significantly increased in RA-SFs, which were reversed almost to those observed in normal SFs after BPNSs treatment (Fig. 5D and 5E). It is noteworthy that the extracellular level of IL-1β, as detected by ELISA, was slightly increased (Fig. 5F), in contrast to TNF-α (Fig. 5G). This suggests that BPNSs selectively interfere with the classical ER-Golgi secretory pathway regulating TNF-α release, rather than the unconventional inflammasome-mediated pathway responsible for IL-1β secretion. In addition, the anti-inflammatory effects of BPNSs were further evidenced by a reduction in oxidative stress (Fig. 5H and 5I), which can be induced by pro-inflammatory cytokines [54].

**Figure 6.**
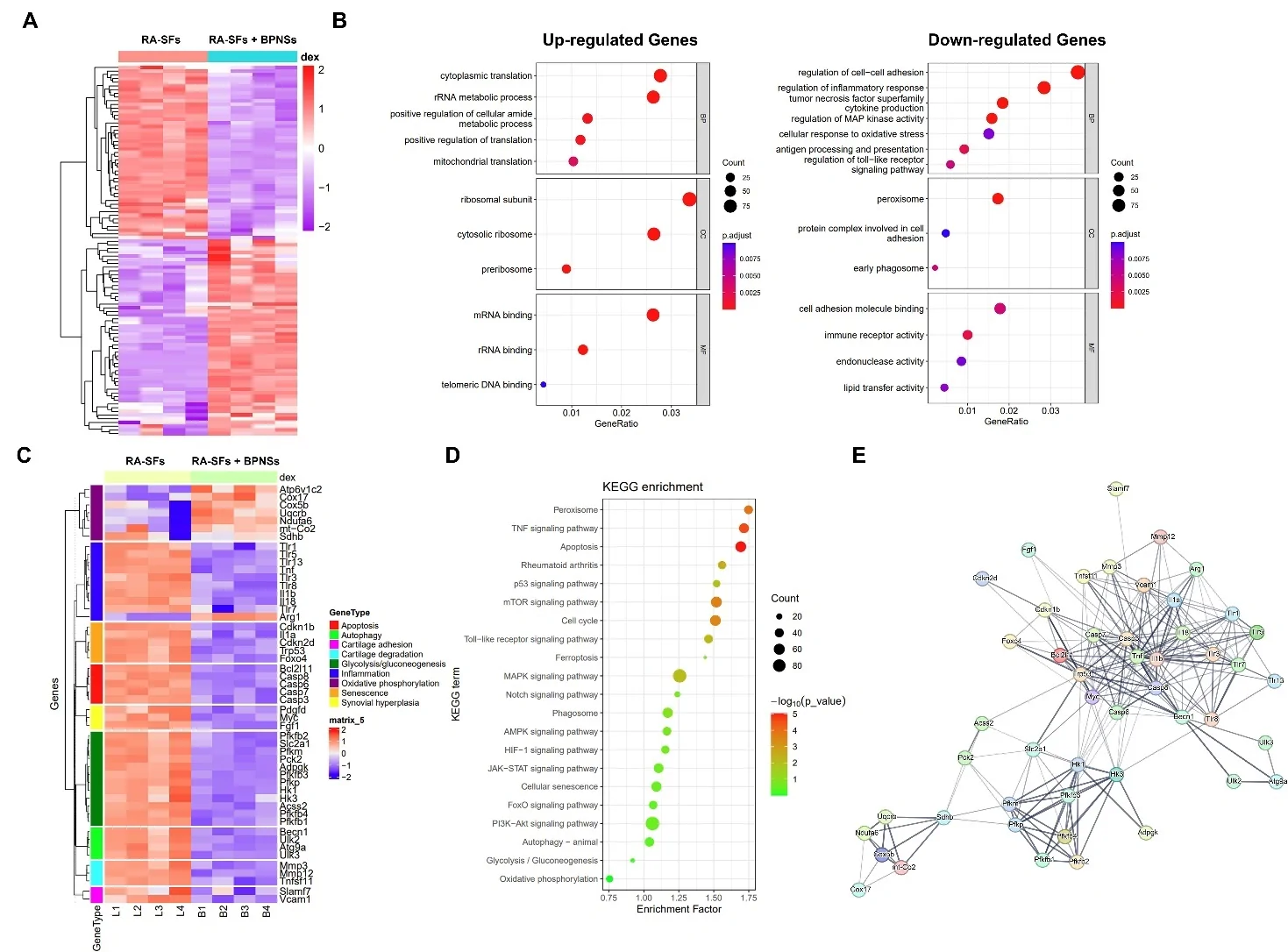
**Functional enrichment analyses of DEGs between untreated and BPNSs-treated RA-SFs**. (**A**) Representative DEGs between untreated and BPNSs-treated RA-SFs. (**B**) GO enrichment analysis of up-regulated and down-regulated DEGs. (**C** and **D**) Heatmap and KEGG pathway enrichment analysis of DEGs between untreated and BPNSs-treated RA-SFs. (**E**) The PPI network of DEGs shown in (C).

### The mechanism of the cytoprotective effects of BPNSs on RA-SFs

To further elucidate the cytoprotective effects of BPNSs on RA-SFs, we performed RNA sequencing on untreated and BPNSs-treated RA-SFs. A total of 7324 differentially expressed genes (DEGs) were identified after BPNSs treatment, with 4330 showing up-regulation and 2994 showing down-regulation compared to untreated controls (Fig. 6A). Consistent with the inhibitory effects of BPNSs on autophagy and apoptosis, down-regulated DEGs included pro-apoptotic genes (*Bcl2l11* [55], *Casp3* [56], *Casp6* [57], *Casp7* [58] and *Casp8* [59]) and pro-autophagic genes (*Atg9a*, *Ulk2*, *Ulk3*, *Becn1*) [60, 61] (Fig. 6C). Meanwhile, genes associated with inflammation activation (*Tnf*, *Il1b*, *Il18*, *Tlr1*, *Tlr3*, *Tlr5*, *Tlr7*, *Tlr8*, *Tlr13*) [62, 63], synovial hyperplasia (*Myc* [64], *Fgf1* [65], *Pdgfd* [66]), cartilage adhesion (*Vcam1*, *Slamf7* (also known as *Cs1* or *Cracc* or *Cd319*)) [64], and cartilage degradation (*Mmp3* [64], *Mmp12* [67], *Tnfsf11* [64]) were down-regulated (Fig. 6C). In contrast, *Arg1*, an anti-inflammatory gene was upregulated. Moreover, SASP-related genes were also down-regulated, including *Foxo4* [68], *Il1a* [69], *Trp53* [70], *Cdkn1b* [71], *Cdkn2d* [72] (Fig. 6C), further confirming the cytoprotective effects of BPNSs. In line with the increased ATP level in RA-SFs after BPNSs treatment, the DEGs were closely associated with energy metabolism. Specifically, up-regulated DEGs included key genes involved in oxidative phosphorylation (*Atp6v1c2*, *Cox17*, *Ndufa6*, *Uqcrb*, *Cox5b*, *mt-Co2*, *Sdhb*) [73-76], while down-regulated DEGs were represented by genes associated with glycolysis/gluconeogenesis (*Pfkfb1* [77], *Pfkfb2* [78], *Pfkfb3* [79], *Pfkfb4* [80], *Acss2* [81], *Adpgk* [82], *Hk3* [83], *Pfkm* [84], *Pfkp* [85], *Pck2* [86], *Hk1* [87], *Slc2a1*(also known as *Glut1*) [88]) (Fig. 6C). This suggests that BPNSs can shift the metabolic pathway of RA-SFs towards oxidative phosphorylation. Supporting this, the lactate level in RA-SFs, indicating aerobic glycolysis, was significantly reduced after BPNSs treatment (Supplementary Fig. 4).

Next, Gene Ontology (GO) and Kyoto Encyclopedia of Genes and Genomes (KEGG) pathway enrichment analyses were conducted to unveil the key biological processes and signaling pathways involving DEGs induced by BPNSs treatment. On the one hand, the GO terms associated with up-regulated DEGs were represented by mitochondrial translation, positive regulation of cellular amide metabolic process, rRNA metabolic process, cytosolic ribosome, and pre-ribosome (Fig. 6B). As these cellular activities are known to contribute to the synthesis of macromolecules, their concomitant up-regulation aligned with our observation of the activation of the anabolic promoter mTOR following BPNSs treatment. On the other hand, the down-regulated DEGs were primarily associated with oxidative stress and inflammatory regulation, including the regulation of inflammatory response, tumor necrosis factor superfamily cytokine production, the regulation of MAP kinase activity, antigen processing and presentation, and the regulation of toll-like receptor signaling pathway (Fig. 6B). The downregulation of these biological processes confirmed the protective effects of BPNSs on RA-SFs against inflammation. Consistently, KEGG pathway analysis showed that DEGs between untreated and BPNSs-treated RA-SFs were enriched in key pathways involved in inflammation, including MAPK signaling pathway, TNF signaling pathway, JAK-STAT signaling pathway, and Toll-like receptor signaling pathway (Fig. 6D). Additionally, Search Tool for the Retrieval of Interacting Genes (STRING) analysis was performed to construct the protein-protein interaction (PPI) network for DEGs shown in Fig. 6C. *Tnf* and *Il1b* were identified as the hub genes (Fig. 6E), indicating that inflammation-related pathways might be the pivotal intersections regulated by BPNSs. These pathways may mediate the crosstalk between different pathways associated with autophagy, apoptosis, aging, and energy metabolism during BPNSs treatment.

### BPNSs induce apoptosis and suppress pro-inflammatory M1 phenotype in BMDMs

**Figure 7.**
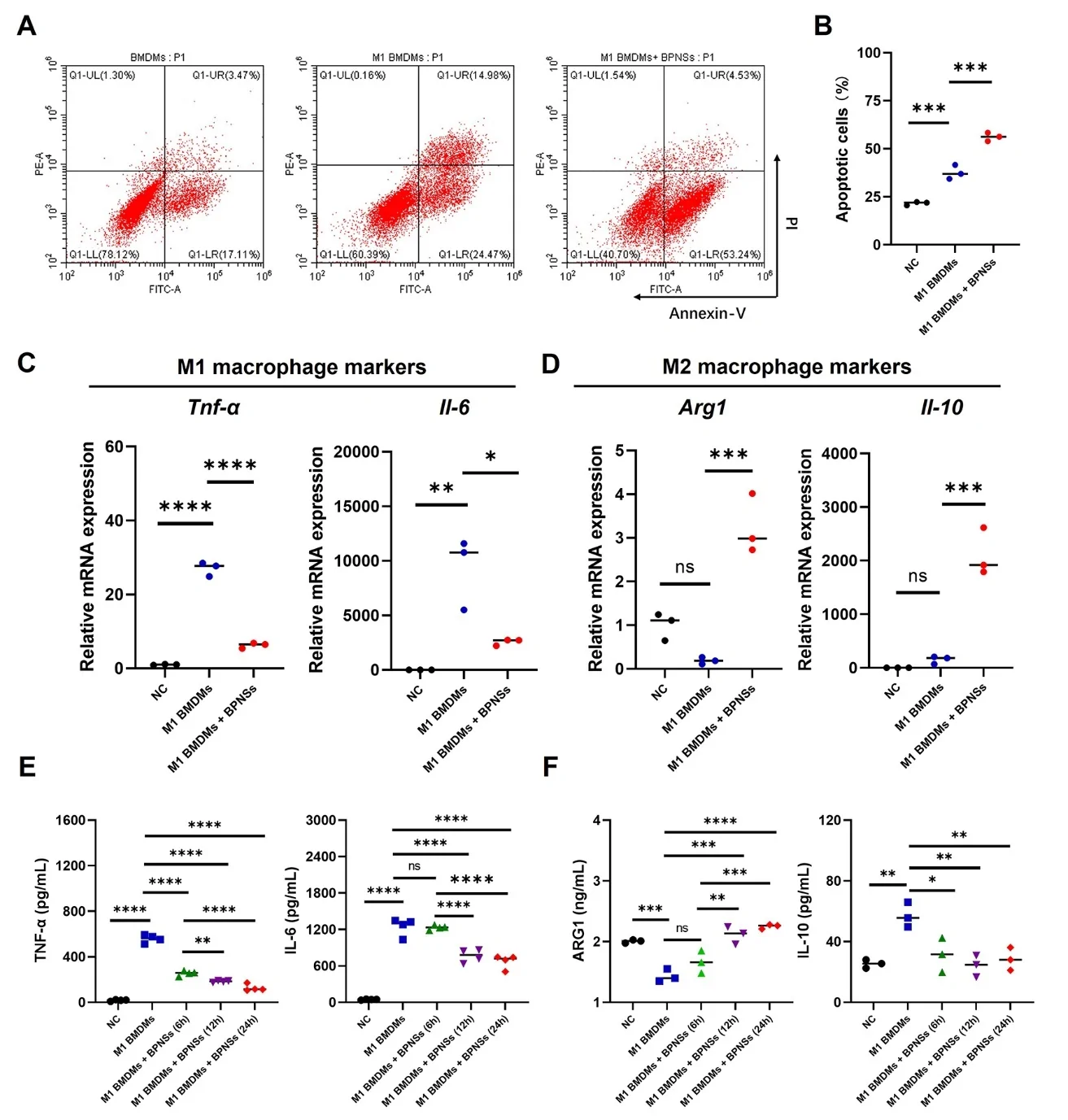
**BPNSs treatment induces the apoptosis of M1 BMDMs and changes the expression of M1 and M2 macrophage markers**. (**A**) Representative flow cytometry plots and (**B**) quantification of apoptotic cells in control cells (NC), M1 BMDMs and BPNSs-treated M1 BMDMs. The apoptotic cells included those in the early (Q1-LR) and late (Q1-UR) stages of apoptosis. (**C** and **D**) Relative mRNA levels of *Tnf-α*, *Il-6*, *Arg1* and *Il-10* in control cells (NC), M1 BMDMs and BPNSs-treated M1 BMDMs. (**E** and **F**) Quantification of TNF-α, IL-6, ARG1 and IL-10 released from control cells (NC), M1 BMDMs and BPNSs-treated M1 BMDMs. For the BPNSs-treated group, the culture media were collected for ELISA analysis at 6 h, 12 h and 24 h after initiating BPNSs treatment. For (B-D and F), n=3 batches of cells per group, indicating 3 biological replicates. For (E), n=4 batches of cells per group, indicating 4 biological replicates. The normality of data was evaluated with the Shapiro-Wilk test. The significance of difference was determined through one-way ANOVA with Tukey's *post hoc* test. Significance levels are denoted as *p < 0.05, **p < 0.01, ***p < 0.001, ****p < 0.0001 and ^ns^p: not significant. NC, negative control, normal BMDMs without LPS induction.

Macrophages are associated with excessive immune activation [89] and the induction of macrophage apoptosis is regarded as an effective strategy in RA treatment. Given the anti-inflammatory properties of BPNSs, we further investigated their therapeutic effects in primary BMDMs [90]. Activated macrophages (M1 BMDMs) were induced by LPS (200 ng/mL, 4 h). After BPNSs treatment, we found a surprisingly high level of apoptosis (Fig. 7A, 7B and Supplementary Fig. 5) accompanied with a significant increase in *caspase-3* expression (Supplementary Fig. 6) in M1 BMDMs. This apoptosis-promoting effect of BPNSs was also observed in normal BMDMs (Supplementary Fig. 7 and Supplementary Fig. 8). Furthermore, BPNSs treatment enhanced the accumulation of ROS in M1 BMDMs and normal BMDMs (Supplementary Fig. 9 and Supplementary Fig.10). This is different from the reduction of ROS levels by BPNSs in RA-SFs, which is attributed to the phagocytotic capacity of BMDMs as immune cells [91]. Indeed, BPNSs uptake by SFs or RA-SFs was obviously lower than that by BMDMs or M1 BMDMs (Supplementary Fig.11). These observations align with a previous study reporting that the high uptake of BPNSs by macrophages led to elevated oxidative stress levels and eventual cell death [92], resembling one of the mechanisms by MTX in treating RA.

Notably, BPNSs treatment significantly reduced the levels of pro-inflammatory cytokines (TNF-α and IL-6) in M1 BMDMs (Fig. 7C and 7E), while increasing the expression of anti-inflammatory molecules (*Il-10* and *Arg1*) linked to the M2 phenotype (Fig. 7D). The different regulation of IL-10 at the mRNA and protein levels (Fig. 7D and 7F) does not contradict each other, as IL-10 can be both anti-inflammatory and pro-inflammatory in specific contexts [93]. Therefore, BPNSs treatment inhibited the pro-inflammatory activity of M1 BMDMs and potentially promoted their transition to a more reparative M2 state. It can also be speculated that the apoptosis-promoting effects of BPNSs in normal BMDMs were selectively targeted towards those with the M1 phenotype.

### Biosafety assessment of BPNSs *in vivo*

Subsequently, we evaluated the biocompatibility of BPNSs *in vivo*. Histopathological images demonstrated that major organs, including the heart, liver, spleen, lung and kidney, experienced negligible changes in mice administrated with BPNSs (5 mg/kg), compared with the control mice (Supplementary Fig. 12A). The liver and kidney are major accumulation sites in the metabolic processes of almost all nanomaterials. Hepatic and renal function parameters, including alanine transaminase (ALT), aspartate aminotransferase (AST), urea, and creatinine (CREA), remained within the normal range after BPNSs treatment and showed no significant changes compared with the controls (Supplementary Fig. 12B). These results indicate that BPNSs have a high level of biosafety *in vivo*.

### Therapeutic Effects of BPNSs on RA *in vivo*

**Figure 8.**
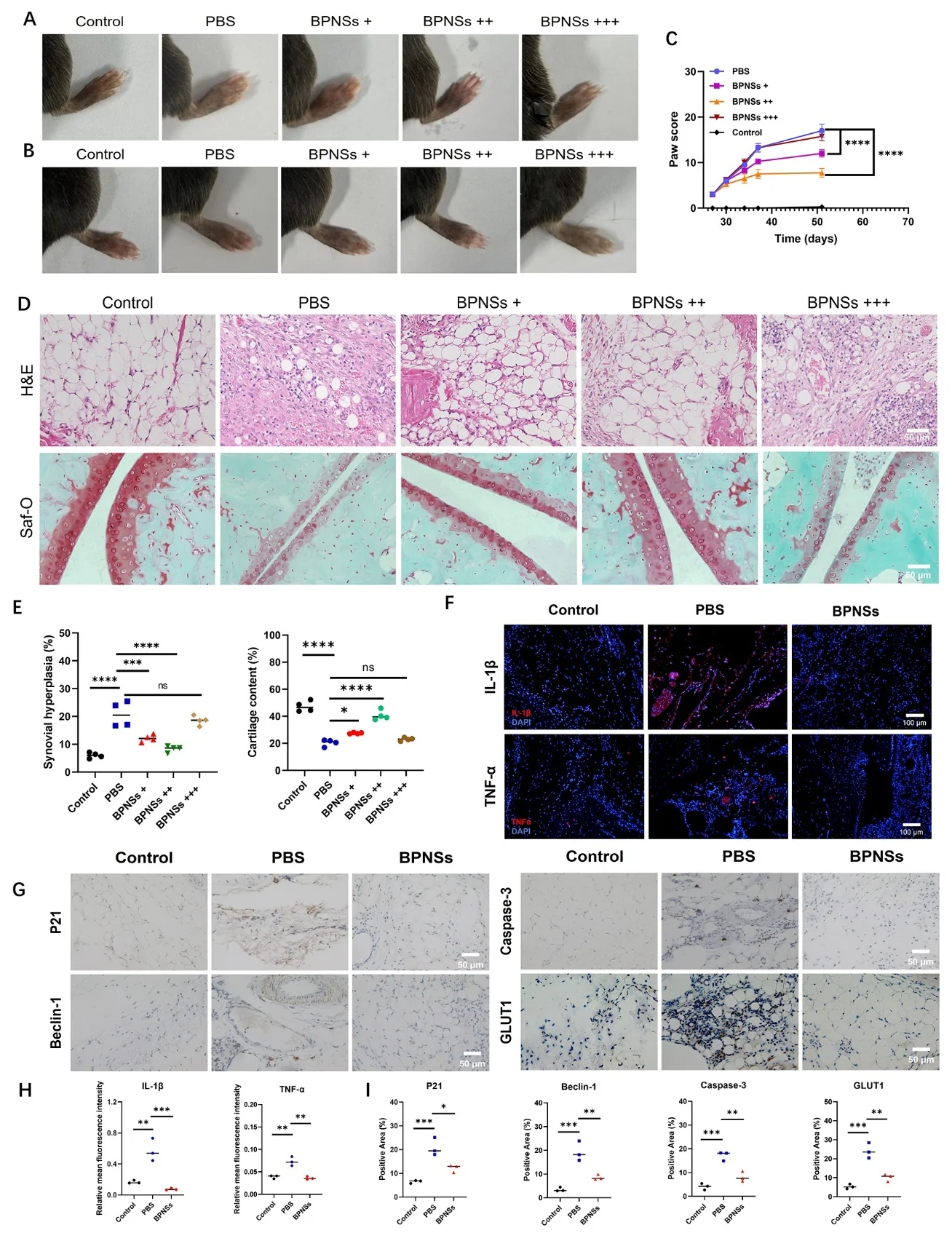
**Therapeutic effects of BPNSs on CIA mice**. (**A** and **B**) Representative photos of the paws of mice with different treatments for 48 h and 51 days, respectively. The wild-type mice and CIA mice injected with PBS were used as the negative control and positive control for RA-related pathology, respectively. (**C**) The cumulative paw scores of mice with different treatments over a 51-day course. (**D**) Representative H&E staining and Saf-O staining images of ankle joints from mice with different treatments. Scale bar, 50 μm. (**E**) Quantification of synovial hyperplasia and cartilage content represented by (D). (**F** and **H**) Representative immunofluorescence staining images and quantification of IL-1β, TNF-α in the ankle joints of mice with different treatments. Scale bar, 100 μm. The nuclei were stained with DAPI. (**G** and **I**) Representative DAB staining images and quantification of P21, caspase-3, Beclin-1 and GLUT1 in the ankle joints of mice with different treatments. Scale bar, 50 μm. For (C and E), n=4 mice per group, indicating 4 biological replicates. For (H and I), n=3 mice per group, indicating 3 biological replicates. The normality of data was evaluated with the Shapiro-Wilk test. The significance of difference was determined through one-way ANOVA with Tukey's *post hoc* test. Significance levels are denoted as *p < 0.05, **p < 0.01, ***p < 0.001, ****p < 0.0001 and ^ns^p: not significant. Control, wild-type mice.

To validate the therapeutic effects of BPNSs *in vivo*, the CIA mice were generated as reported previously [35]. BPNSs were administered via intra-articular injection every three days for two weeks to three experimental groups: the BPNSs + group (0.2 mg/kg), BPNSs ++ group (0.5 mg/kg) and BPNSs +++ group (1.2 mg/kg). As early as 48 h after BPNSs treatment, significant relief in paw swelling was observed in the BPNSs ++ group (Fig. 8A). On day 51, the PBS-treated CIA mice showed a significant increase in the cumulative paw swelling score compared with the control group, while the BPNSs + and BPNSs ++ groups exhibited significant improvements, with the BPNSs ++ group demonstrating the optimal therapeutic outcome (Fig. 8B and 8C). On the contrary, the BPNSs +++ group showed no significant improvement (Fig. 8B and 8C), highlighting the importance of BPNSs dosage. The therapeutic effect of BPNSs was further evaluated by histological analysis. H&E staining and Saf-O staining of the ankle joints respectively showed alleviation of synovial hyperplasia and cartilage damage in the BPNSs + and BPNSs ++ groups (Fig. 8D and 8E). This is consistent with the down-regulation of genes associated with synovial hyperplasia, cartilage adhesion and cartilage degradation (Fig. 6C). Similarly, these healing effects were not observed in the BPNSs +++ group.

The BPNSs ++ group with the most significant therapeutic effects was selected for additional evaluations through immunofluorescence staining and immunohistochemical staining. It was found that the enhanced expression of TNF-α and IL-1β, two key cytokines involved in the development of RA, was significantly down-regulated in the ankle joints of CIA mice treated with BPNSs (Fig. 8F and 8H), further validating their anti-inflammatory role. Significantly, the expression levels of caspase-3, Beclin-1, P21 and GLUT1 were also reduced following BPNSs treatment (Fig. 8G and 8I). These results aligned with the downregulation of pathways related to apoptosis, autophagy, cellular senescence, and aerobic glycolysis observed in RA-SFs treated with BPNSs (Fig. 6B and 6C).

## DISCUSSION

In the progression of RA, SFs and synovial macrophages are impaired by external and internal stimuli. RA-SFs appear to be more resistant to apoptosis than osteoarthritis synovial fibroblasts (OA-SFs) due to overactivated autophagy [94]. Meanwhile, synovial macrophages of M1 phenotype exacerbate the progression of RA. While temporary inflammation activates autophagy and immune responses to clear harmful factors, persistent stress disrupts homeostasis and impairs the self-repair capacity of the organism, ultimately leading to chronic diseases [95]. Overactivated autophagy and immune responses, two prominent features of RA, have been identified as important targets of RA treatment strategies [96]. Combining these therapeutic concepts, we synthesized BPNSs and demonstrated their capability to inhibit autophagy while exerting cytoprotective effects in RA-SFs. While showing excellent biocompatibility with SFs, BPNSs may induce apoptosis in BMDMs and decrease the expression levels of cytokines associated with the pro-inflammatory M1 phenotype. To our knowledge, few single nanomaterials exhibit multiple therapeutic effects similar to BPNSs. The straightforward preparation process of BPNSs also makes them suitable for clinical applications.

Previous studies have shown that antigen-presenting cells, including dendritic cells, macrophages, and activated B cells initiate immune responses and thereby play a role in autoimmune diseases [97]. Massive pro-inflammatory cytokines may cause synovial hyperplasia and bone injury, ultimately leading to joint destruction [98]. While commonly used DMARDs reduce synovitis and delay joint destruction, they have common drawbacks, such as long half-lives and accumulation with continued use, which contribute to a high risk of side effects [99]. Emerging treatment strategies PDT and PTT take advantage of the physical properties of nanomaterials to elevate local tissue temperature and increase the level of ROS upon light irradiation in order to kill pro-inflammatory cells in RA microenvironment [100, 101]. However, the limited penetration efficacy of light poses challenges in achieving the desired therapeutic effects [102], therefore constraining their clinical applicability. Certain types of nanomedicines for RA, such as noble metal nanoparticles, metal oxide nanomaterials, carbon-based and silicon-based nanomaterials, are known to have low biocompatibility due to their stable properties [101, 103-105]. Consequently, eliminating these materials from the body can be challenging. By contrast, the distinctive degradation behavior of BPNSs ensures rapid excretion. In this study, we verify that BPNSs possess excellent biocompatibility both *in vitro* and *vivo*, as evidenced by a nearly 100% cell viability of SFs treated with 200 μg/mL BNPSs and marginal changes in major organs of mice after BPNSs administration (5 mg/kg).

RA-SFs produce pro-inflammatory cytokines and matrix metalloproteinases (MMPs) that promote bone destruction in the joints of RA patients, and thus actively contribute to the progression of RA [106]. Activated autophagy pathways have been reported in the synovial tissues of RA patients and are strongly correlated with the severity of the disease [107]. Furthermore, autophagy has been suggested to facilitate the presentation of citrullinated peptides to T cells, incurring chronic inflammation [108]. Here, our findings reveal that BPNSs can inhibit autophagy at an early stage via the suppression of autophagosome formation in RA-SFs, effectively protecting the cells against apoptosis and inflammation. This is in line with the favorable biocompatibility of BPNSs, as the inhibition of autophagy at a late stage potentially causes the accumulation of autophagosomes and results in cytotoxicity [109]. Notably, instead of inducing RA-SFs to enter the apoptotic cycle, BPNSs act by repairing these cells. We propose that the degradation product of BPNSs, PO_4_^3-^, facilitates ATP synthesis. The subsequent increase in intracellular ATP level sends a "nutrient sufficiency" signal to RA-SFs, leading to decreased AMPK activity, which in turn lifts the inhibition on mTOR signaling pathway, resulting in the down-regulation of autophagy regulatory complex and autophagosome biogenesis [48].

RA-SFs are known to undergo a shift of glucose metabolism from oxidative phosphorylation to aerobic glycolysis, similar to tumor-like cells [110, 111]. The aerobic glycolysis, also known as the Warburg effect, is marked by the up-regulation of hexokinase 2 (HK2) and GLUT1, and contributes to a more inflammatory phenotype of RA-SFs [112], exacerbating the symptoms of RA. Accordingly, the pro-inflammatory effects can be suppressed by glycolysis inhibitors [113]. Our results demonstrate that BPNSs treatment can restore the energy metabolic pathway of RA-SFs to oxidative phosphorylation. This regulatory mechanism may rectify harmful metabolic imbalance and bring the cells back to a normal state.

Promoting macrophage apoptosis and decreasing the expression levels of M1 phenotype-related cytokines have been confirmed as an effective therapeutic strategy for RA [114]. However, depleting macrophages may delay or compromise tissue healing [115]. BPNSs deliver selective killing of M1 BMDMs while promoting their transition to the M2 phenotype, effectively addressing concerns related to excessive macrophage exhaustion. Furthermore, in more severe systemic immune diseases, such as lupus erythematosus [116], the selective toxicity of BPNSs to M1 macrophages may offer new therapeutic strategies.

Importantly, *in vivo* evaluations confirm the therapeutic effects of BPNSs on RA, as evidenced by the amelioration in paw swelling, synovial hyperplasia, synovial inflammation, and cartilage damage. However, several questions remain to be addressed before BPNSs can be put to clinical use. Firstly, it is noteworthy that the dosage of BPNSs appears to influence their therapeutic outcome in CIA mice. To maximize their therapeutic efficacy for clinical use, it is necessary to optimize the concentration using cells, tissues or organoids derived from RA patients. Secondly, the administration route of BPNSs in RA patients is crucial for the drug's efficacy, distribution, and safety profile. In response to this consideration, the microneedle system has been suggested to deliver RA-treating nanomedicine due to its advantages of precise drug delivery to damaged tissue, bypassing the circulatory system and healthy tissue, and preventing overdose [117]. Lastly, while the fast degradation property of BPNSs greatly reduces the possibility of side effects, it poses a challenge for large-scale production or long-term use. To address this issue, we propose using BPNSs in combination with less-traumatic phototherapies, such as PTT and PDT to achieve synergistic treatment of RA. Altogether, our study identifies BPNSs as a promising new drug for RA treatment and provides a comprehensive explanation of the underlying therapeutic mechanism, as well as highlights the broader biomedical application values of BPNSs in chronic inflammatory and age-related diseases.

## Supplementary Data

The Supplementary data can be found online at: www.aginganddisease.org/EN/10.14336/AD.2024.0319.

## Data Availability

The data reported in this manuscript and supplementary files are available from the corresponding authors upon reasonable request.
