# Supplementary material for: Treatment of Rheumatoid Arthritis Based on the Inherent Bioactivity of Black Phosphorus Nanosheets
Source: Aging Dis. 2024 Jun 3;16(3):1652–73. doi: 10.14336/AD.2024.0319 (PMC12096912; doi:10.14336/AD.2024.0319)
Supplement: Supplementary file 1 — The Supplementary data can be found online at: www.aginganddisease.org/EN/10.14336/AD.2024.0319. [file AD-16-3-1652-s.pdf]

SUPPLEMENTARY DATA

Treatment of Rheumatoid Arthritis Based on the Inherent Bioactivity of Black Phosphorus Nanosheets

Cheng Zhuang, Ruiqi Sun, Yuchen Zhang, Qing Zou, Jianxin Zhou, Naijun Dong, Xuyu Zhao, Wenjun Fu, Xiaoke Geng, Jiao Wang, Qian Li, Robert Chunhua Zhao

© 2024. Zhuang C et al. Published online at http://www.aginganddisease.org/EN/10.14336/AD.2024.0319

SUPPLEMENTARY DATA

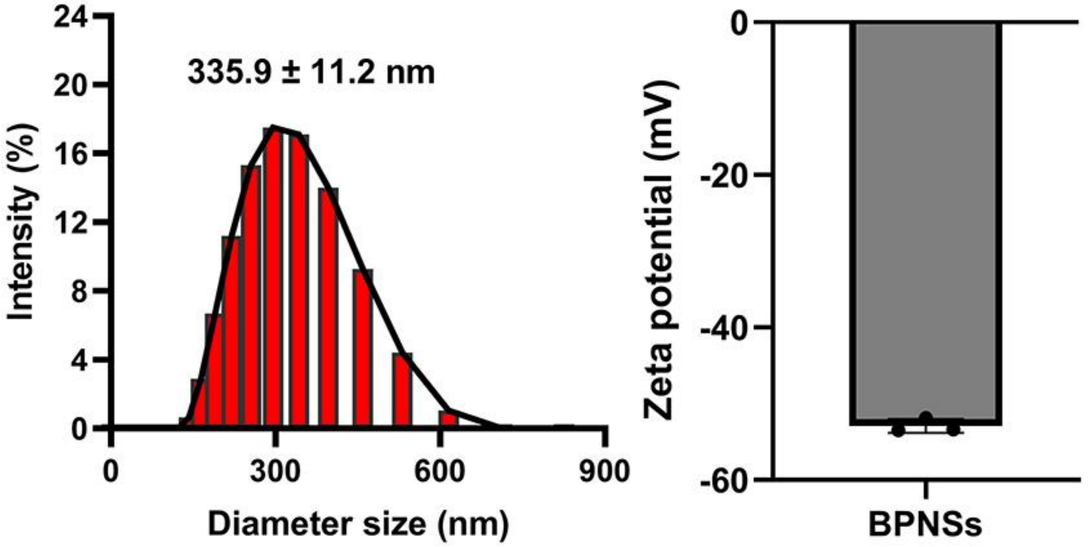

Supplementary Figure 1. Size distribution and zeta potential of BPNSs are determined by DLS.

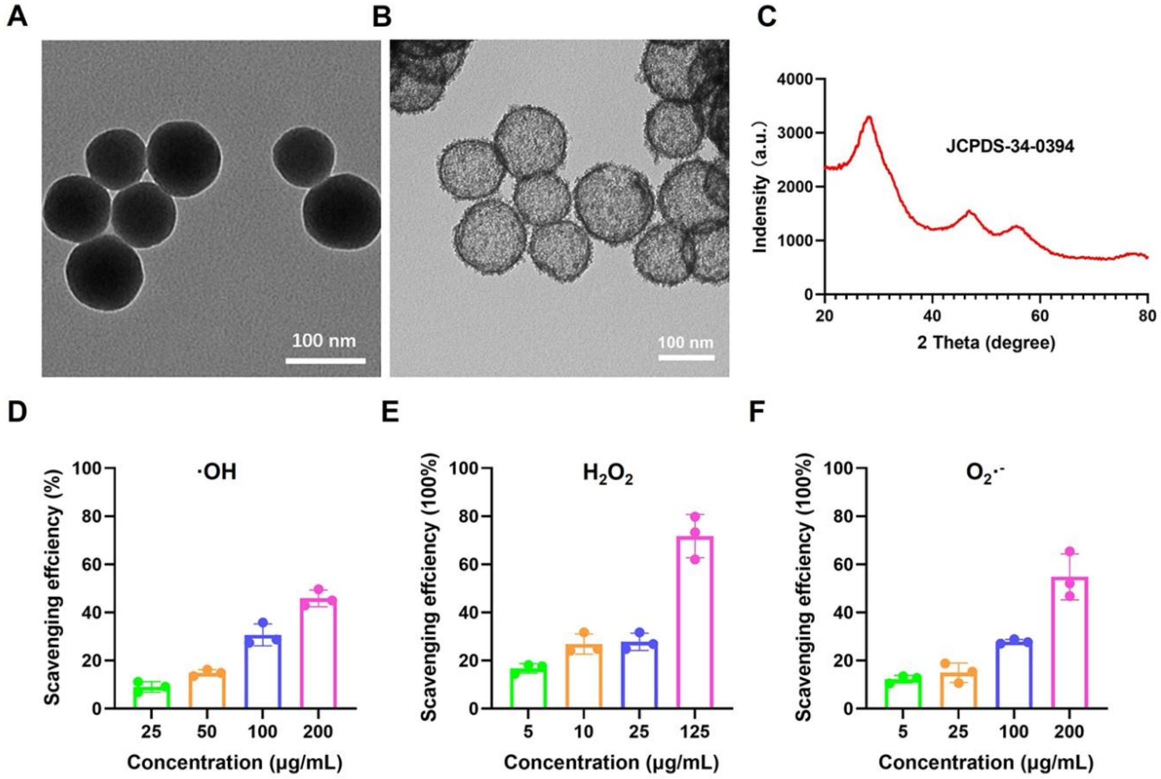

Supplementary Figure 2. Characterization and antioxidant capacities of $HCeO_2$ NPs. (**A**, **B**) Representative TEM images of (**A**) SiO_2 NPs and (**B**) $HCeO_2$ NPs. (**C**) The X-ray diffraction spectrum of $HCeO_2$ NPs. (**D-F**) The scavenging rates of $HCeO_2$ NPs for (**D**) ·OH, (**E**) H_2O_2 and (**F**) $O_2{}^{\cdot -}$. For (D-G), n=3 independent experiments, indicating 3 biological replicates.

© 2024. Zhuang C et al. Published online at http://www.aginganddisease.org/EN/10.14336/AD.2024.0319

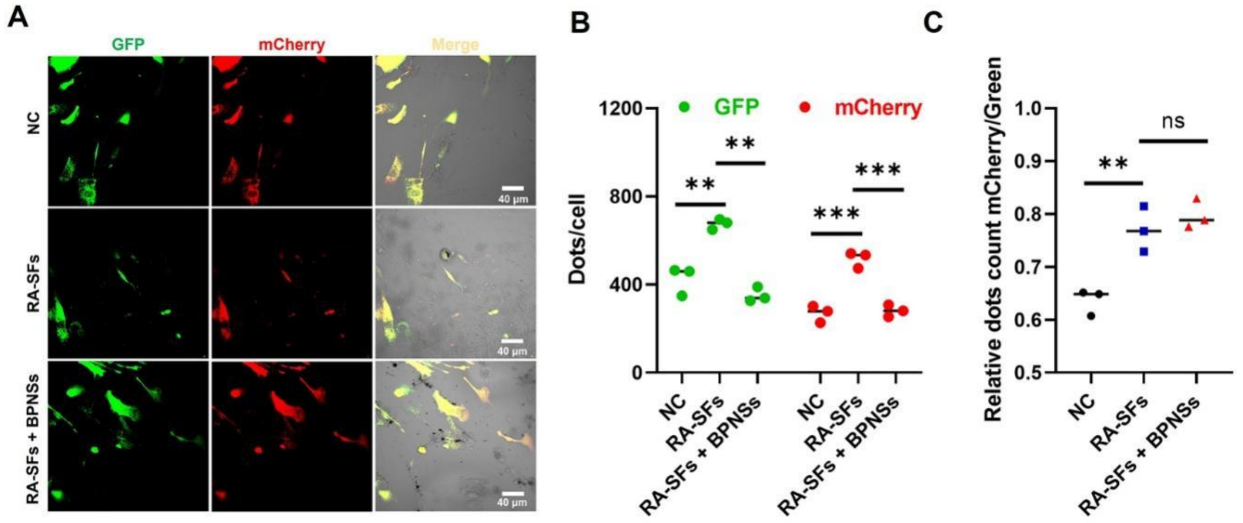

Supplementary Figure 3. Tracking of the autophagic process in control cells (NC), RA-SFs and BPNSs-treated RA-SFs. (**A**) Representative confocal microscopy images of mCherry-GFP-LC3B expression in control cells (NC), RA-SFs and BPNSs-treated RA-SFs. (**B**, **C**) Quantification of green dots, red dots, and the ratio of green to red dots represented by (A). The co-labeling of GFP and mCherry indicates the presence of early-stage autophagosomes, while the single labeling of mCherry represents late-stage autolysosomes. Scale bar, 40 μm. N=3 batches of cells per group. The normality of data was evaluated with the Shapiro-Wilk test. Significance of difference was determined through one-way ANOVA with Tukey's *post hoc* test. Significance levels are denoted as **p < 0.01, ***p < 0.001, and ^{ns}p: not significant. NC, negative control, normal SFs without LPS induction.

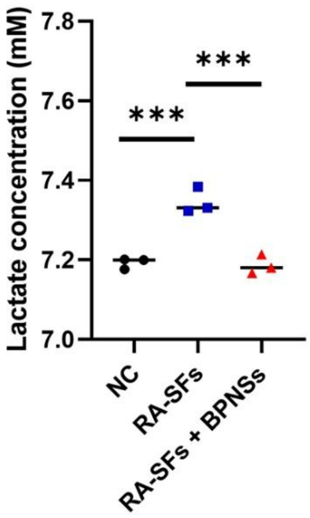

Supplementary Figure 4. The lactate levels in control cells (NC), RA-SFs and BPNSs-treated RA-SFs. N=3 batches of cells per group. The normality of data was evaluated with the Shapiro-Wilk test. The significance of difference was determined through one-way ANOVA with Tukey's *post hoc* test. The significance level is denoted as ***p < 0.001. NC, negative control, normal SFs without LPS induction.

© 2024. Zhuang C et al. Published online at http://www.aginganddisease.org/EN/10.14336/AD.2024.0319

SUPPLEMENTARY DATA

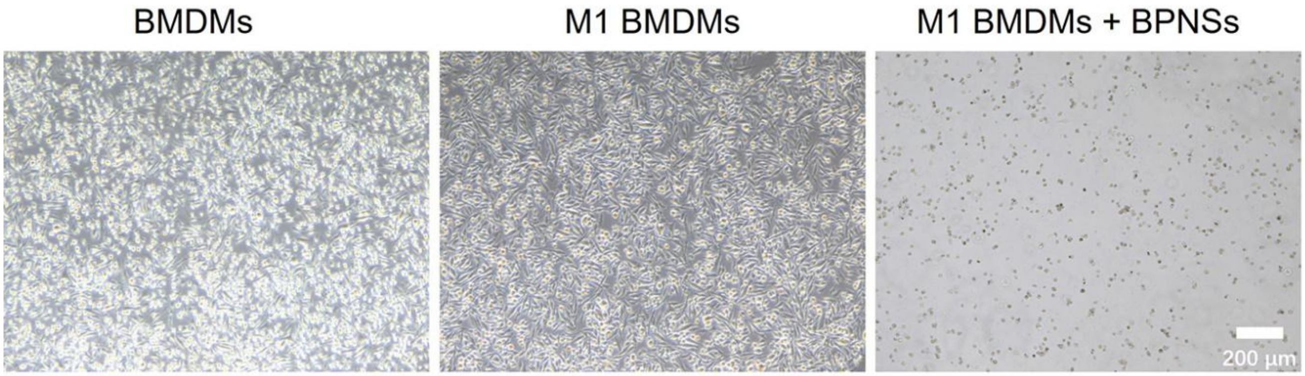

Supplementary Figure 5. Representative bright-field images of normal BMDMs, M1 BMDMs and BPNSs-treated M1 BMDMs. Scale bar, 200 µm.

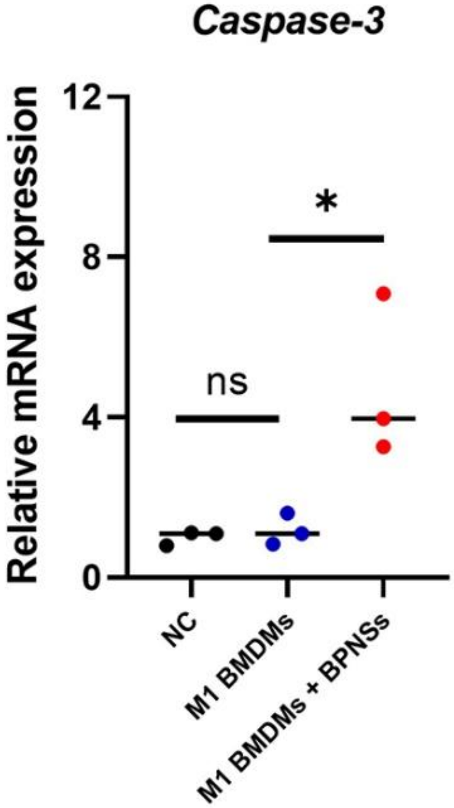

Supplementary Figure 6. The mRNA expression levels of *caspase-3* in control cells (NC), M1 BMDMs and BPNSs-treated M1 BMDMs. N=3 batches of cells per group. The normality of data was evaluated with the Shapiro-Wilk test. The significance of difference was determined through one-way ANOVA with Tukey's *post hoc* test. Significance levels are denoted as *$p < 0.05$ and ^{ns}p: not significant. NC, negative control, normal BMDMs without LPS induction.

© 2024. Zhuang C et al. Published online at http://www.aginganddisease.org/EN/10.14336/AD.2024.0319

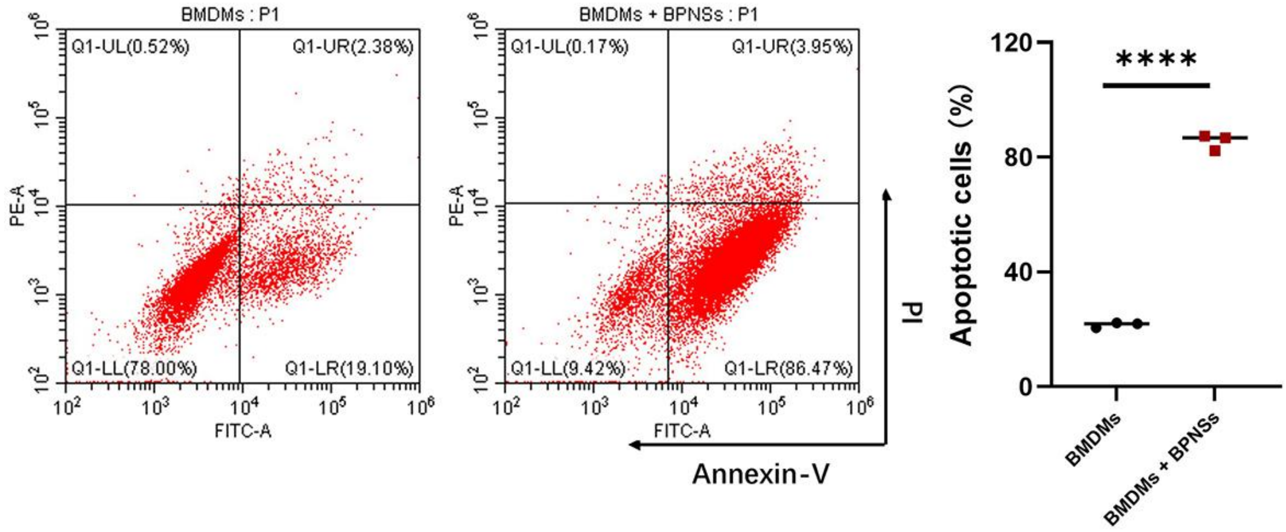

Supplementary Figure 7. Representative flow cytometry plots and quantification of apoptotic BMDMs treated with or without BPNSs. The apoptotic cells included those in the early (Q1-LR) and late (Q1-UR) stages of apoptosis. N=3 batches of cells per group. The normality of data was evaluated with the Shapiro-Wilk test. The significance of difference was determined through Student's t-test. The significance level is denoted as ****p < 0.0001.

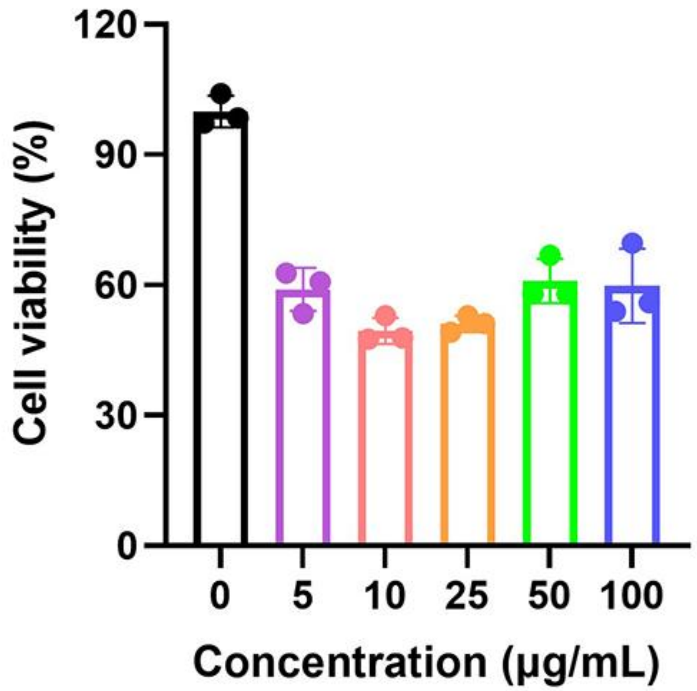

Supplementary Figure 8. Cell viability of BMDMs treated with different concentrations of BPNSs. The values were quantified with a CCK-8 kit from 3 independent replicates (n=3).

© 2024. Zhuang C et al. Published online at http://www.aginganddisease.org/EN/10.14336/AD.2024.0319

SUPPLEMENTARY DATA

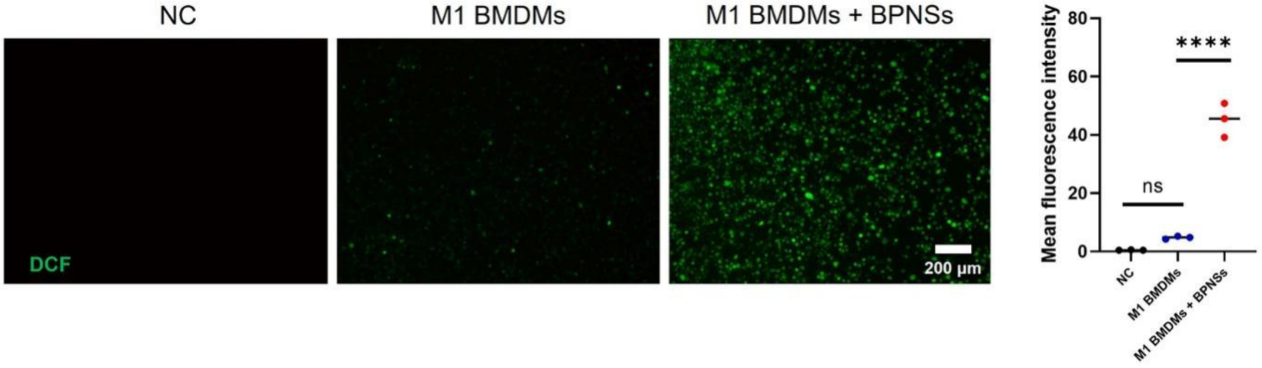

Supplementary Figure 9. Representative immunofluorescence images and quantification of intracellular ROS levels in control cells (NC), M1 BMDMs and BPNSs-treated M1 BMDMs. The ROS level is represented by the average fluorescence intensity of DCF labeling. N=3 batches of cells per group, indicating 3 biological replicates. The normality of data was evaluated with the Shapiro-Wilk test. The significance of difference was determined through one-way ANOVA with Tukey's *post hoc* test. Significance levels are denoted as ****$p < 0.0001$ and ^{ns}p: not significant. Scale bar, 200 µm. NC, negative control, normal BMDMs without LPS induction.

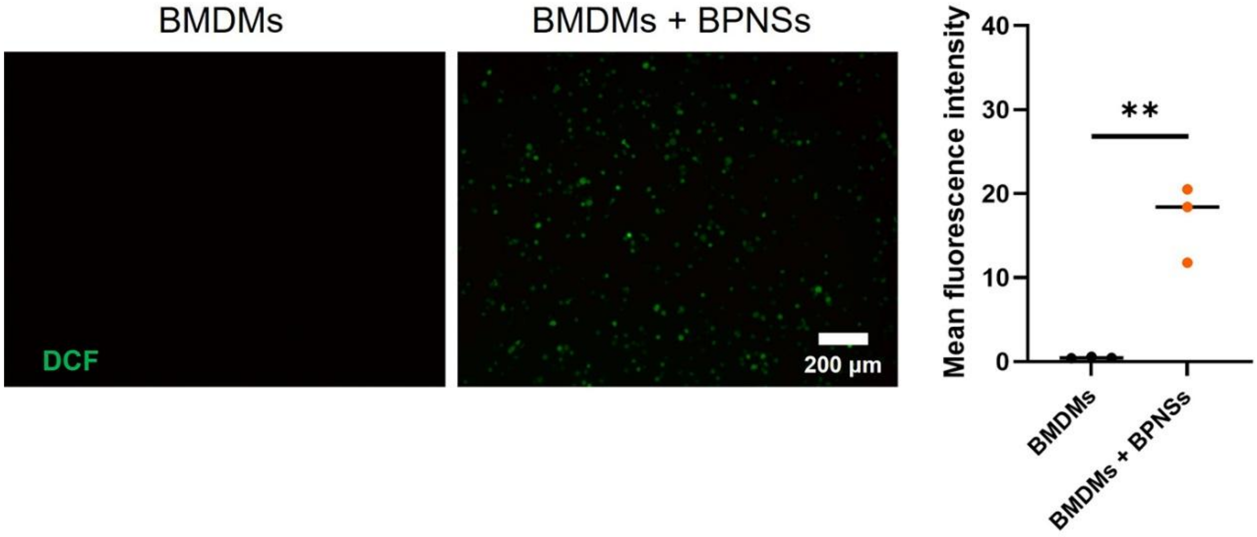

Supplementary Figure 10. Representative immunofluorescence images and quantification of intracellular ROS levels in BMDMs treated with or without BPNSs. The ROS level is represented by the fluorescence intensity of DCF labeling. N=3 batches of cells per group, indicating 3 biological replicates. The normality of data was evaluated with the Shapiro-Wilk test. The significance of difference was determined through Student's t-test. The significance level is denoted as **$p < 0.01$. Scale bar, 200 µm.

© 2024. Zhuang C et al. Published online at http://www.aginganddisease.org/EN/10.14336/AD.2024.0319

SUPPLEMENTARY DATA

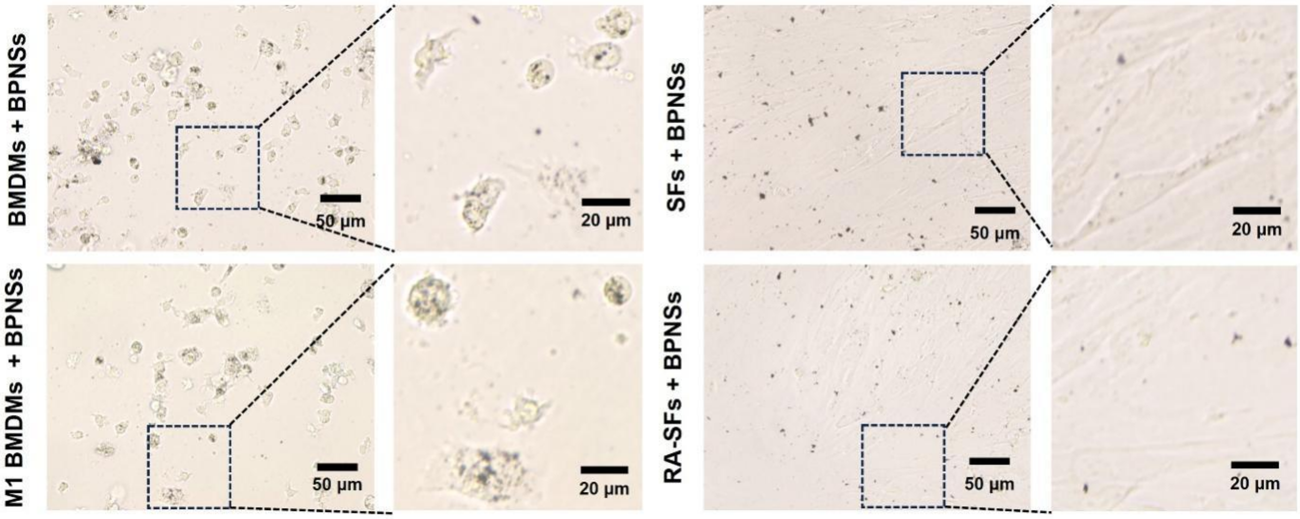

Supplementary Figure 11. Representative bright-field images of BMDMs, M1 BMDMs, SFs and RA-SFs treated with BPNSs respectively. The black dots represent BPNSs. Scale bar, 50 μm and 20 μm.

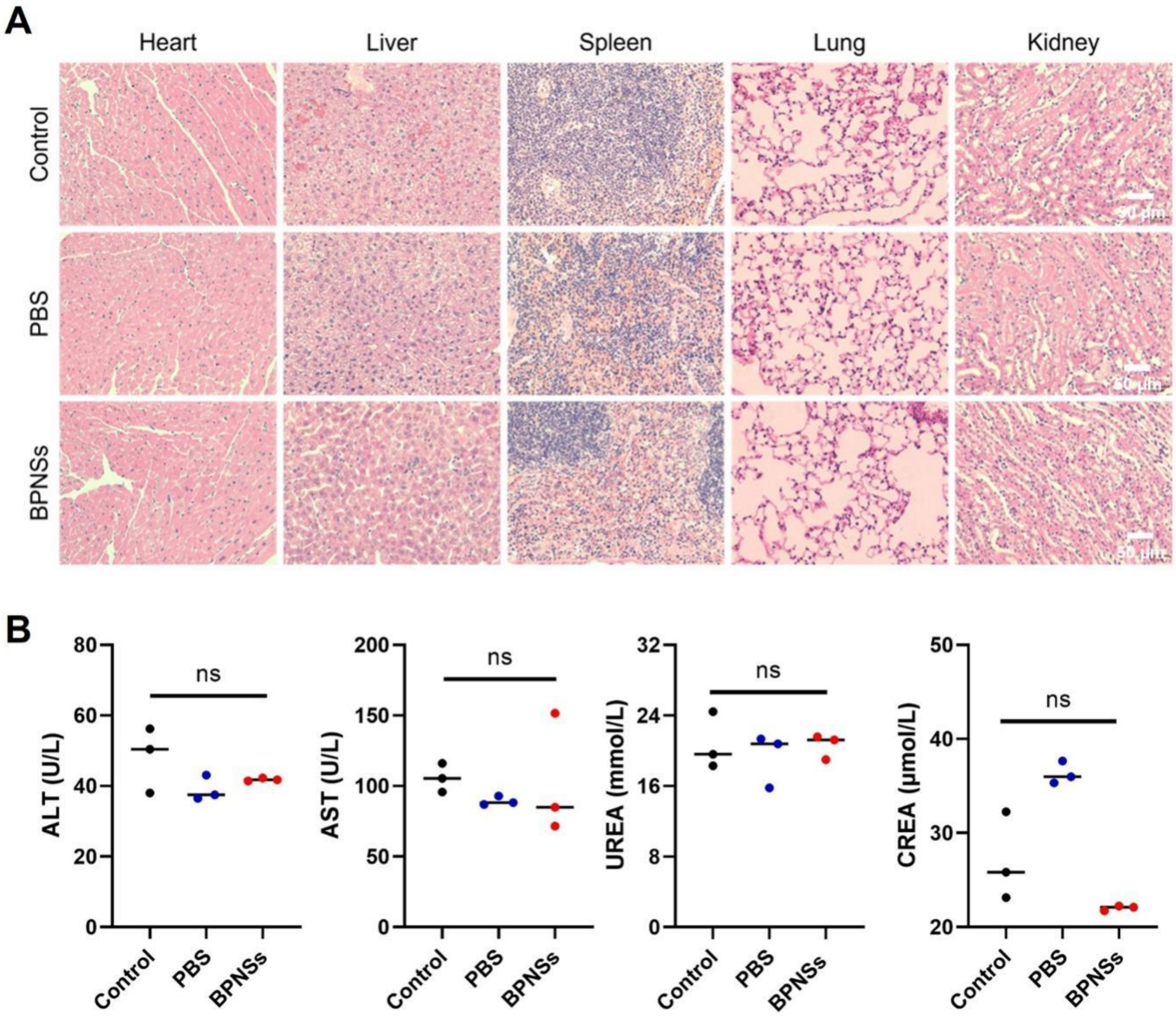

© 2024. Zhuang C et al. Published online at http://www.aginganddisease.org/EN/10.14336/AD.2024.0319

SUPPLEMENTARY DATA

Supplementary Figure 12. Biosafety assessment of BPNSs *in vivo*. (**A**) Representative H&E staining images of major organs (heart, liver, spleen, lung, and kidney) from control mice and CIA mice with or without BPNSs treatment (5 mg/kg). Scale bar, 50 μm. The wild-type mice and CIA mice injected with PBS were used as the negative control and positive control for RA-related pathology, respectively. (**B**) Quantification of renal and hepatic parameters of mice described in (A). The values were quantified from 3 mice per group (n=3). The normality of data was evaluated with the Shapiro-Wilk test. Significance of difference was determined through one-way ANOVA. The significance level is denoted as ^{ns}p: not significant.

© 2024. Zhuang C et al. Published online at http://www.aginganddisease.org/EN/10.14336/AD.2024.0319
